# SYGL-1 and LST-1 link niche signaling to PUF RNA repression for stem cell maintenance in *Caenorhabditis elegans*

**DOI:** 10.1371/journal.pgen.1007121

**Published:** 2017-12-12

**Authors:** Heaji Shin, Kimberly A. Haupt, Aaron M. Kershner, Peggy Kroll-Conner, Marvin Wickens, Judith Kimble

**Affiliations:** 1 Department of Biochemistry, University of Wisconsin-Madison, Madison, Wisconsin, United States of America; 2 Howard Hughes Medical Institute, University of Wisconsin-Madison, Madison, Wisconsin, United States of America; University of Minnesota, UNITED STATES

## Abstract

Central questions in regenerative biology include how stem cells are maintained and how they transition from self-renewal to differentiation. Germline stem cells (GSCs) in *Caeno-rhabditis elegans* provide a tractable *in vivo* model to address these questions. In this system, Notch signaling and PUF RNA binding proteins, FBF-1 and FBF-2 (collectively FBF), maintain a pool of GSCs in a naïve state. An open question has been how Notch signaling modulates FBF activity to promote stem cell self-renewal. Here we report that two Notch targets, SYGL-1 and LST-1, link niche signaling to FBF. We find that SYGL-1 and LST-1 proteins are cytoplasmic and normally restricted to the GSC pool region. Increasing the distribution of SYGL-1 expands the pool correspondingly, and vast overexpression of either SYGL-1 or LST-1 generates a germline tumor. Thus, SYGL-1 and LST-1 are each sufficient to drive “stemness” and their spatial restriction prevents tumor formation. Importantly, SYGL-1 and LST-1 can only drive tumor formation when FBF is present. Moreover, both proteins interact physically with FBF, and both are required to repress a signature FBF mRNA target. Together, our results support a model in which SYGL-1 and LST-1 form a repressive complex with FBF that is crucial for stem cell maintenance. We further propose that progression from a naïve stem cell state to a state primed for differentiation relies on loss of SYGL-1 and LST-1, which in turn relieves FBF target RNAs from repression. Broadly, our results provide new insights into the link between niche signaling and a downstream RNA regulatory network and how this circuitry governs the balance between self-renewal and differentiation.

## Author summary

Stem cells lie at the heart of metazoan development, regeneration, and tissue homeostasis, but the molecular basis of their regulation is poorly understood in their natural context within an animal. Here we investigate this problem in the nematode gonad, where germline stem cells are maintained by Notch signaling from the niche and PUF RNA binding proteins in stem cells. Yet the link between Notch and PUF has been elusive. The two Notch target genes essential for GSC maintenance encode novel proteins with few clues to function. Here we report that these mysterious proteins are cytoplasmic and function post-transcriptionally as PUF partners to ensure RNA repression. We also show that the restricted spatial distribution of these newly identified regulators governs the size of the stem cell pool and prevents tumor formation. In sum, our results demonstrate how niche signaling is linked with downstream regulators to govern the stem cell fate and establish a stem cell pool.

## Introduction

The balance between stem cell self-renewal and differentiation is pivotal for normal development, adult homeostasis, and regeneration. Indeed, aberrant stem cell regulation can cause disease, including human degenerative disorders and cancers [1]. Stem cell daughters can exist in a “naïve” multipotent state or a “primed” state that has been triggered to differentiate, typically via transit-amplification [2–4]. Stem cells that divide asymmetrically rely on oriented cell division to generate one naïve and one primed daughter [e.g. 5], but the mechanism underlying stem cells that divide stochastically to generate pools of naïve and primed daughters [e.g. 6, 7] remains largely unanswered. Challenges have included the complexity of their niches [8] and diversity of stem cell states (e.g. quiescent vs. proliferative) [9]. Thus, understanding how stem cell daughters are regulated to remain naïve or transition to a primed state can greatly benefit from a tractable model with well-defined niche and stem cells.

**Fig 1:**
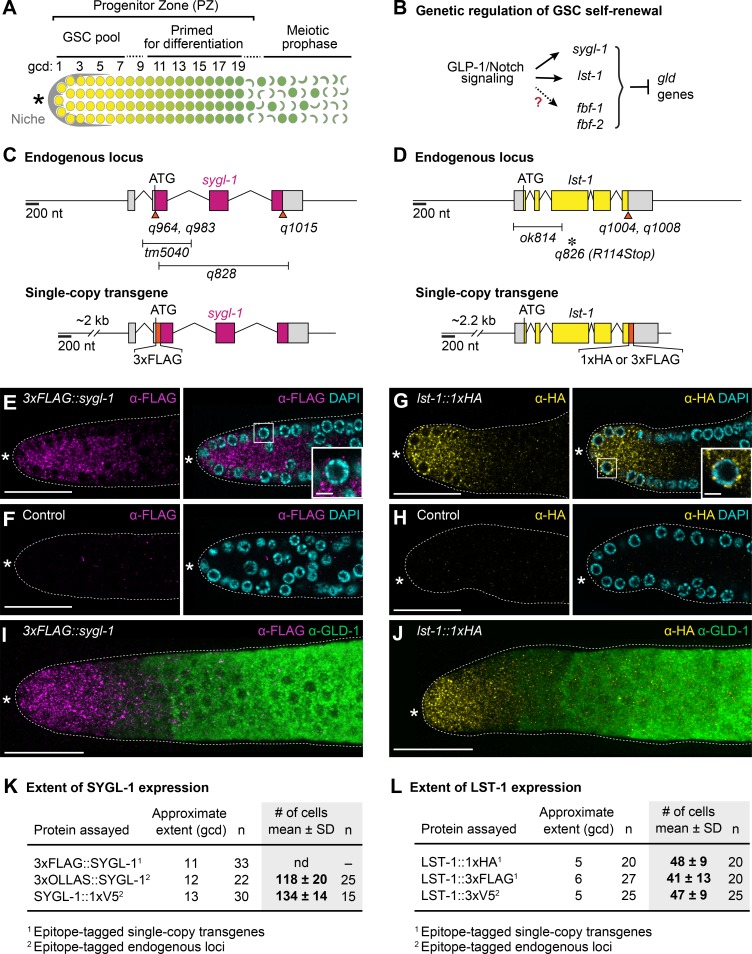
SYGL-1 and LST-1 proteins are spatially restricted to the GSC pool region. (A) Schematic of adult distal gonad. The progenitor zone (PZ) includes a distal pool of germline stem cells (GSC) and a proximal pool of cells primed to differentiate [11]. The conventional metric for axis position is number of germ cell diameters from the distal end (gcd). Somatic niche for GSCs (gray); naïve stem cell state (yellow circles); early meiotic prophase (green crescents); primed transiting state (yellow to green gradient). Asterisk marks distal end. (B) Genetic pathway of GSC regulation. (C and D) Schematics of *sygl-1 and lst-1* loci (top) and transgenes (bottom). Epitope tagged endogenous alleles are: *sygl-1(q964)[3xMYC*::*sygl-1]*, *sygl-1(q983)[3xOLLAS*::*sygl-1]* and *sygl-1(q1015)[sygl-1*::*1xV5]*; *lst-1(q1004)[lst-1*::*3xV5]* and *lst-1(q1008)[lst-1*::*3xOLLAS]*. Colored boxes, *sygl-1* or *lst-1* exons; gray boxes, untranslated regions; orange boxes and triangles, epitopes. Bars below schematic, deletions; asterisk, nonsense mutation. See Methods for updated gene structures. (E-J) SYGL-1 and LST-1 proteins in dissected adult gonads. (E-H) Representative slice or (I-J) maximum intensity z-projections of distal gonad stained with α-FLAG (SYGL-1, magenta), α-HA (LST-1, yellow), α-GLD-1 (green), and DAPI (cyan). Dashed line, gonadal outline; asterisk, distal end. Scale bar is 20 μm in all images, except 5 μm in (E) and (G) insets. (E) *sygl-1(q828); qSi49[P*_*sygl-1*_::*3xFLAG*::*sygl-1*::*sygl-1 3’end]*. (F) *sygl-1(q828)*. (G) *lst-1(ok814); qSi22[P*_*lst-1*_::*lst-1*::*1xHA*::*lst-1 3’end]*. (H) *lst-1(ok814)*. See **S1A–S1C Fig** for whole gonad images. (K and L) Extent of SYGL-1 and LST-1 expression along the gonadal axis, estimated with functional epitope-tagged proteins. Expression is robust distally and graded proximally. Proximal boundaries were estimated by eye as the point at which staining becomes barely detected. nd, not determined. See **S1D and S1E Fig** for data supporting functionality of epitope-tagged proteins and see **S2 Fig** for characterization of *sygl-1* or *lst-1* mutants.

The *Caenorhabditis elegans* gonad provides a paradigm for analyzing regulation of a stem cell pool [10]. In this system, a single-celled mesenchymal niche maintains a pool of ~225 stochastically-dividing germ cells in the “progenitor zone” (**Fig 1A**) [10]. That progenitor zone itself consists of a distal pool of 30–70 naïve germline stem cells (GSCs) and a more proximal pool of GSC daughters that have been triggered to begin differentiation and hence have been “primed” (**Fig 1A**) [11]. Central to GSC maintenance are two conserved regulators, Notch signaling and PUF (for Pumilio and FBF) RNA-binding proteins [12, 13]. GLP-1/Notch signaling from the niche is essential for GSC maintenance [14] and two nearly identical PUF proteins, FBF-1 and FBF-2 (collectively FBF), act as broad-spectrum repressors of differentiation RNAs to promote GSC self-renewal (**Fig 1B**) [15, 16]. FBF provides one regulatory hub in the stem cell regulatory network; other hubs rely on GLD translational regulators to drive differentiation [17]. However, key questions remain. Here we focus on how Notch signaling and FBF repression are coordinated to establish a naïve GSC pool and facilitate transition to the primed state.

Recently-identified GSC regulators are the *sygl-1* and *lst-1* genes, which are direct targets of niche signaling [18]. The *lst-1 sygl-1* double mutant exhibits the same severe GSC loss as a GLP-1/Notch mutant while single mutants maintain GSCs, revealing functional redundancy [18]. However, the molecular functions of SYGL-1 and LST-1 have been a mystery. LST-1 harbors a single Nanos-like zinc finger, suggesting a possible role in post-transcriptional regulation. Yet both proteins are composed largely of low-complexity regions; neither is recognizable beyond Caenorhabditids; and the two amino acid sequences bear little resemblance to each other despite their redundancy [18]. Despite the novelty of these proteins, their striking GSC loss phenotype coupled with the restriction of their mRNAs to a region corresponding to the GSC pool [18, 19] suggested that understanding their function and regulation would provide insights into regulation of a stem cell pool.

Here we investigate SYGL-1 and LST-1 proteins to understand their roles in stem cell regulation. We find that both are cytoplasmic proteins and spatially restricted to the GSC region. Intriguingly, modest SYGL-1 expansion increases size of the stem cell pool, and vast expansion of either SYGL-1 or LST-1 drives formation of a germline tumor. The SYGL-1 and LST-1–dependent tumors form in the absence of GLP-1/Notch signaling, reinforcing their key roles in stem cell maintenance. However, SYGL-1 and LST-1 no longer drive tumor formation in the absence of FBF. Consistent with the idea that SYGL-1 and LST-1 drive stem cell self-renewal in a complex with FBF, SYGL-1 and LST-1 interact physically with FBF and are required for repression of an FBF target RNA. We suggest that SYGL-1 and LST-1 are FBF partners and function to ensure repression of FBF target RNAs within the stem cell pool.

## Results

### SYGL-1 and LST-1 are restricted to the GSC pool region

To visualize SYGL-1 and LST-1 proteins, we generated epitope-tagged versions of *sygl-1* and *lst-1*, including single-copy transgenes using MosSCI [20–22] and endogenous alleles using CRISPR-Cas9 [23, 24] (**Fig 1C and 1D**). Importantly, these epitope-tagged SYGL-1 and LST-1 proteins were functional: they maintain GSCs when tested in appropriate mutant backgrounds (**S1D and S1E Fig**). Therefore, they mimic their wild-type counterparts and we refer to them henceforth as SYGL-1 and LST-1. By immunostaining, both proteins were expressed in the cytoplasm of the distal-most germ cells within the progenitor zone**:** SYGL-1 was largely punctate while LST-1 was enriched in perinuclear granules (**Figs 1E–1H** and **S1A–S1C**). Using the conventional metric for position along the gonadal axis, germ cell diameters (gcd) from the distal end (**Fig 1A**), we found SYGL-1 enriched from 1-~12 gcd, and LST-1 from 1-~5 gcd (**Fig 1K and 1L**, see legend for how we determined extents). These protein extents correspond well to the distributions of their respective wild-type mRNAs, assayed by single-molecule FISH [19], and were reproducible regardless of epitope tag. We counted the number of germ cells stained for each protein and found SYGL-1 in ~125 cells and LST-1 in ~45 germ cells (**Fig 1K and 1L**). Strikingly, high SYGL-1 and LST-1 levels were correlated with low GLD-1 expression (**Fig 1I and 1J**), consistent with their opposing functions (see Introduction). We conclude that SYGL-1 and LST-1 are restricted within the progenitor zone to the GSC region, consistent with their pivotal roles in GSC self-renewal.

### Moderate expansion of SYGL-1 expands GSC pool size moderately

The spatial restriction of SYGL-1 and LST-1 proteins suggested that their distribution might govern size of the GSC pool. Previous studies reported that progenitor zones (PZ) were smaller in *sygl-1* and *lst-1* single mutants than in wild type [18, 25], but GSC pool size was not analyzed. We first confirmed the decreased PZ size in mutants used previously, *sygl-1(tm5040)* and *lst-1(ok814)*. We also generated additional mutants: *sygl-1(q828)* deletes the entire open reading frame plus all introns (**Fig 1C**) and *lst-1(q826)* harbors a premature stop codon (**Fig 1D**). PZ sizes were essentially the same for the various alleles of each gene (**S2A and S2B Fig**), as were other measures (e.g. brood size, fertility, embryonic lethality) (**S2C and S2D Fig**), suggesting that all are strong loss-of-function. We call them *sygl-1(0)* and *lst-1(0)* henceforth. Consistent with previous results [18, 25], the PZ size was affected differently for the two genes: the *sygl-1(0)* PZ was about half the size of wild type, while the *lst-1(0)* PZ was only marginally smaller than wild type (**S2A and S2B Fig**). We therefore focused on the SYGL-1 extent and its relationship to GSC pool size.

**Fig 2:**
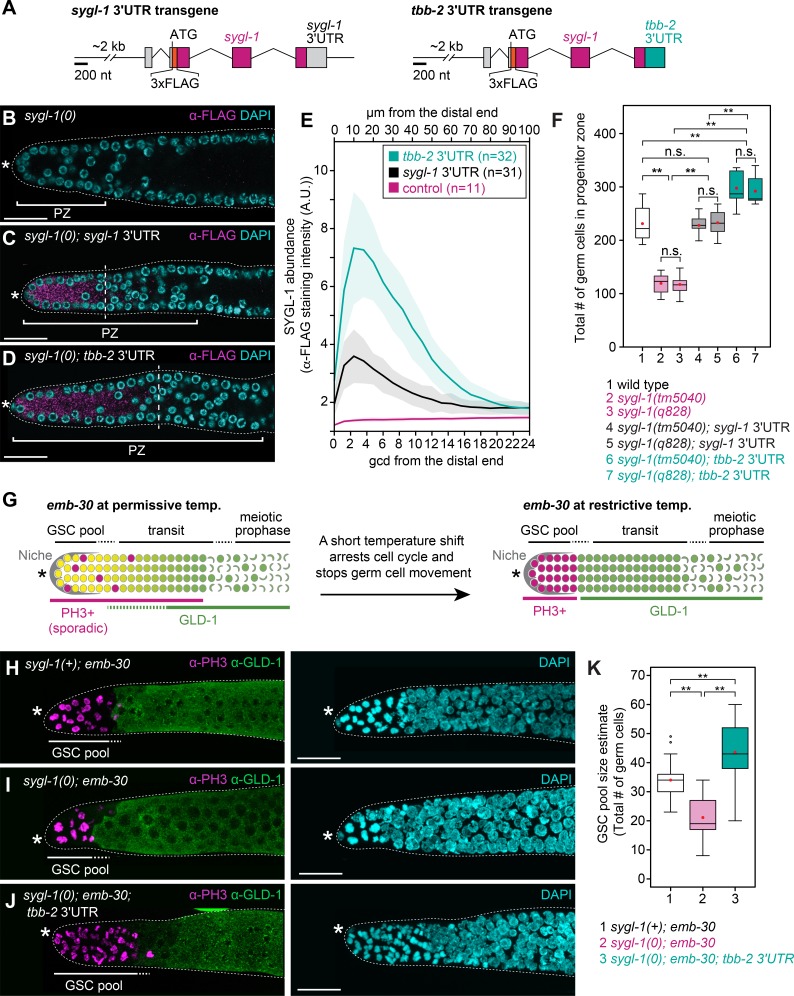
Extent of SYGL-1 expression domain correlates with size of GSC pool. (A) Schematics of transgenes. Conventions as in **Fig 1C**. Left, *sygl-1* 3’UTR transgene. Right, *tbb-2* 3’UTR transgene replaces *sygl-1* 3’UTR with *tbb-2* (β-tubulin) 3’UTR. See **S3 Fig** for data supporting functionality of *tbb-2* 3’UTR transgene. (B-D) Extents of SYGL-1 protein in dissected adult gonads stained with α-FLAG (SYGL-1, magenta) and DAPI (cyan). Conventions as in **Fig 1E–1J**; scale bar is 20 μm. (B) *sygl-1(q828)*. (C) *sygl-1(q828); qSi49[P*_*sygl-1*_::*3xFLAG*::*sygl-1*::*sygl-1 3’end]*. (D) *sygl-1(q828); qSi150[P*_*sygl-1*_::*3xFLAG*::*sygl-1*::*tbb-2 3’end]*. (E) Quantitation of SYGL-1 abundance, based on intensity of α-FLAG staining. Average intensity values were plotted against distance in microns along the gonadal axis (x-axis, top), which were converted to the conventional metric of germ cell diameters from distal end (x-axis, bottom) (see Methods). Lines, average intensity in arbitrary units (A.U.); shaded areas, standard deviation; n, number of gonadal arms. (F) Progenitor zone sizes. Averages and standard deviations for each genotype are as follows: (1) 231 ± 33 (n = 12); (2) 119 ± 17 (n = 22); (3) 117 ± 16 (n = 20); (4) 229 ± 16 (n = 15); (5) 234 ± 23 (n = 12); (6) 298 ± 34 (n = 13); (7) 292 ± 25 (n = 12). Bottom and top boundaries of each box, first and third quartiles; middle lines, median; red dots, mean; whiskers, minimum and maximum values. Asterisks indicate a statistically significant difference by Welch’s ANOVA with Games-Howell *post hoc* test. **p<0.001, n.s. = non-significant. (G) *emb-30* assay to measure GSC pool size. An *emb-30* temperature-sensitive mutant stops germ cell movement by cell cycle arrest [29]. At permissive temperature (15°C), the distal gonad appears normal, with scattered PH3-positive M-phase cells and graded GLD-1, a differentiation marker. A shift to restrictive temperature (25°C) reveals a distal pool of naïve stem-like germ cells arrested in M-phase and a proximal pool of germ cells primed to differentiate and hence expressing GLD-1 [11]. (H-J) GSC pool size correlates with SYGL-1 expression. Maximum intensity z-projected images of dissected gonads stained with α-PH3 (magenta), α-GLD-1 (green) and DAPI (cyan). Conventions as in **Fig 1E–1J**; scale bar is 20 μm. (H) Control: *emb-30(tn377ts)*. (I) *sygl-1(tm5040); emb-30(tn377ts)*. (J) *sygl-1(tm5040); qSi150[P*_*sygl-1*_::*3xFLAG*::*sygl-1*::*tbb-2 3’end]; emb-30(tn377ts)*. (K) GSC pool size estimates. Box plot conventions as in **Fig 2F**. Averages and standard deviations for each genotype are as follows: (1) 35 ± 7; (2) 21 ± 7; (3) 43 ± 11; n>28 gonadal arm per genotype. Asterisks indicate a statistically significant difference by 1-way ANOVA with Tukey HSD *post hoc* test. ** p<0.001. Genotypes as in **Fig 2H-2J**.

The onset of SYGL-1 expression relies on Notch signaling from the niche, which activates *sygl-1* transcription [18, 19], but we thought its distribution might be refined post-transcriptionally since genome-wide studies identified RNA regulatory proteins binding to the *sygl-1* 3’UTR [26, 27]. To test this idea, we replaced the *sygl-1* 3’UTR with a 3’UTR that supports expression throughout the germline, the tubulin *tbb-2* 3’UTR [28]. The transgene carrying this 3’UTR replacement was otherwise identical to the *sygl-1* transgene described above (**Fig 1C**), including insertion into the same chromosomal site and rescue of *lst-1 sygl-1* double mutants from sterility to fertility (**S3A Fig**). For simplicity, we refer in this section to the wild-type version as the “*sygl-1* 3’UTR” transgene, and to the replacement version as the “*tbb-2* 3’UTR” transgene (**Fig 2A**). The *tbb-2* 3’UTR transgene, assayed in the absence of endogenous SYGL-1, produced both an expanded distribution of SYGL-1 (~15 gcd or ~1.4-fold more extended than normal) (**Fig 2B–2D**) and more abundant SYGL-1 (~2-fold more than normal) (**Fig 2E**). We conclude that the wild-type *sygl-1* 3’UTR restricts SYGL-1 distribution and lowers its abundance.

We first found that PZ size was 1.3-fold larger in *tbb-2* 3’UTR transgenic animals than in either *sygl-1* 3’UTR transgenic animals or wild type (**Fig 2F**). To test the idea that GSC pool size might also be enlarged, we used the *emb-30* assay [11]. Briefly, this assay arrests cell divisions with a temperature-sensitive allele of *emb-30* (*tn377*), which encodes a component of the anaphase promoting complex [29]. This arrest stops proximal movement of germ cells through the progenitor zone and resolves them into two discrete pools: cells in the distal GSC pool remain naïve and acquire an M-phase marker (PH3), while cells in the proximal pool are primed to differentiate and acquire a differentiation marker (GLD-1) [11] (**Fig 2G**). With this assay, we estimated GSC pool sizes in strains carrying *emb-30* and either the wild-type *sygl-1* locus (normal SYGL-1), the *sygl-1* null mutant (no SYGL-1) or the *tbb-2* 3’UTR transgene (expanded SYGL-1). GSC pools with wild-type SYGL-1 contained ~35 naïve cells; those with no SYGL-1 contained ~21, and those with expanded SYGL-1 had ~43 on average (**Fig 2K**). Indeed, the 1.4-fold increase in SYGL-1 extent (from ~11 to ~15 gcd, on average) corresponds well with the estimated 1.3-fold increase in GSC number (from 35 to 43, on average) and PZ germ cell number (from 229 to 298, on average). Importantly, the extent of LST-1 expression along the gonadal axis (gcd) and number of LST-1–expressing cells in the distal gonad were essentially the same in *sygl-1(+)* and *sygl-1(0)* germlines as well as those harboring the *tbb-2* 3’UTR transgene (**S3B–S3F Fig**). The simplest explanation is that LST-1 expression is likely independent of SYGL-1: The smaller LST-1 expression domain establishes a smaller GSC pool size in *sygl-1* mutants, but that extent of SYGL-1 expression establishes GSC pool size in wild-type and *tbb-2* 3’UTR animals. We conclude that GSC pool size correlates with SYGL-1 extent and suggest that GSC pool size correlates with LST-1 extent in the absence of SYGL-1.

### Ubiquitous germline expression of SYGL-1 or LST-1 generates a tumor

**Fig 3:**
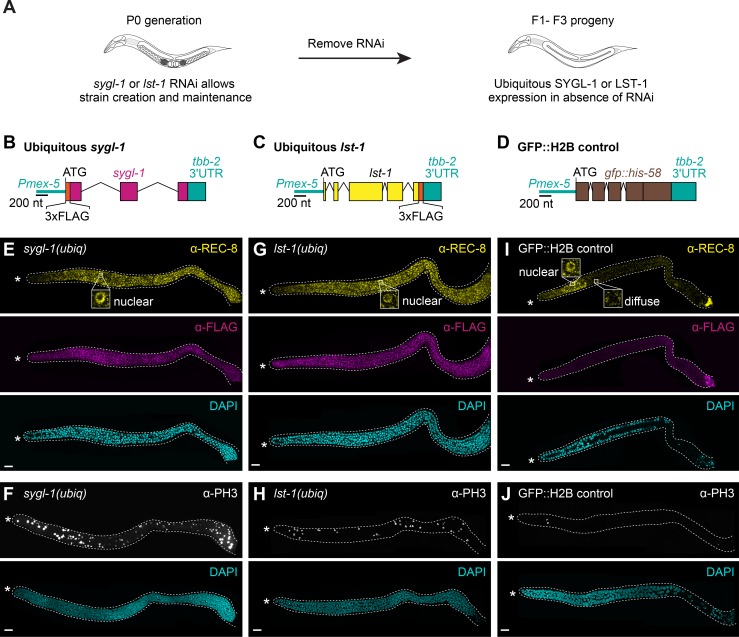
Ubiquitous germline expression of SYGL-1 or LST-1 drives tumor formation. (A) Protocol to induce ubiquitous germline expression of SYGL-1 or LST-1. See text for explanation and **S4A and S4B Fig** for tumor penetrance over generations. (B-D) Schematics of transgenes. The *mex-5* promoter and *tbb-2* 3’UTR were used to promote ubiquitous germline expression. (E-J) Young adult gonads stained with mitotic marker α-REC-8 (yellow), α-FLAG (SYGL-1 or LST-1, magenta), M-phase marker α-PH3 (white), and DAPI (cyan). Images are either single slice (E, G, I) or maximum intensity z-projections (F, H, J). Conventions as in **Fig 1E–1J**; scale bar is 20 μm. (E and F) Genotype for ubiquitous SYGL-1: *sygl-1(tm5040); qSi235[P*_*mex-5*_::*3xFLAG*::*sygl-1*::*tbb-2 3’end]*. (G and H) Genotype for ubiquitous LST-1: *lst-1(ok814); qSi267[P*_*mex-5*_::*lst-1*::*3xFLAG*::*tbb-2 3’end]*. (I and J) Genotype for ubiquitous GFP::H2B control, *weSi2[P*_*mex-5*_::*GFP*::*his-58*::*tbb-2 3’end]* [91]. See **S4C–S4K Fig** for further characterization.

To extend the idea that distributions of SYGL-1 and LST-1 govern GSC pool size, we tested the effect of expressing SYGL-1 or LST-1 throughout the germline. To this end, we made single-copy transgenes driven with a *mex-5* germline promoter and the *tbb-2* 3’UTR, which supports ubiquitous expression throughout the germline [28] (**Fig 3A**). For brevity, we refer to the transgenes as *sygl-1(ubiq)* and *lst-1(ubiq)*, respectively (**Fig 3B and 3C**). Because ubiquitous germline expression of SYGL-1 or LST-1 might render animals sterile, we created transgenes on *sygl-1* or *lst-1* feeding RNAi, and scored effects after RNAi removal, waiting 2–3 generations to minimize transgenerational RNAi inheritance (**Fig 3A**). Strikingly, ubiquitous germline expression of either SYGL-1 or LST-1 created extensive germline tumors (**Fig 3E–3H**). The penetrance of tumor formation depended on both temperature and number of generations after removal from RNAi, but was close to 100% for both *sygl-1(ubiq)* and *lst-1(ubiq)* after two or three generations at 15°C (**S4A and S4B Fig**). About half of these tumors were proliferative throughout the gonad, while the other half included cells in the meiotic cell cycle, perhaps due to incomplete release from RNAi inheritance. Control animals harboring a GFP::H2B transgene driven with the same regulatory elements (**Fig 3D**) had no tumors (**Fig 3I and 3J**), demonstrating that the tumors are specific to SYGL-1 or LST-1.

We next used markers to determine the state of cells in *sygl-1(ubiq)* and *lst-1(ubiq)* tumors. REC-8 localizes to the nucleus of germ cells in the mitotic cycle [30] and REC-8 was nuclear throughout the tumor (**Figs 3E and 3G**, **S4I and S4J**); PH3 marks M-phase [31] and was seen in dividing cells throughout the tumor (**Fig 3F and 3H**); and PGL-1 marks germ cells [32] and also was found throughout the tumor (**S4C and S4D Fig**). Therefore, *sygl-1(ubiq)* and *lst-1(ubiq)* tumors are composed of germ cells that are mostly in the mitotic rather than the meiotic cell cycle. In addition, FBF-1 was abundant and GLD-1 was low throughout the tumors, consistent with germ cells being in an undifferentiated state (**S4F and S4G Fig**). As expected, all markers behaved like wild type in the GFP::H2B control (**Figs 3I and 3J, S4E**, **S4H** and **S4K**).

We also assessed *sygl-1(ubiq)* and *lst-1(ubiq)* tumors for features reported in other mutants with germline tumors. The *sygl-1(ubiq)* and *lst-1(ubiq)* tumors formed in both XX hermaphrodites (**Fig 3E and 3G**) and XO males (**S4I and S4J Fig**), in contrast to hermaphrodite-specific *gld-1* tumors [33]. They formed in animals making only sperm (males) or only oocytes (XX *fog-3* females [34]), in contrast to spermatogenic-specific *puf-8* germline tumors [35]. Finally, they did not rely on Notch signaling (see below), in contrast to tumors arising from inappropriate soma/germline interactions or ectopic Notch activation [e.g. 36–39]. Thus, the most likely explanation of SYGL-1 and LST-1 tumors is that each regulator is sufficient to promote stemness in a germ-cell autonomous fashion and to do so in both sexes.

### Placement of SYGL-1 and LST-1 in the GSC regulatory pathway

The *sygl-1(ubiq)* and *lst-1(ubiq)* strains provide new reagents to explore how SYGL-1 and LST-1 function within the GSC regulatory pathway. Previous analyses placed *sygl-1* and *lst-1* downstream of, or parallel to, GLP-1/Notch signaling and upstream of GLD differentiation regulators, but their relationship with FBF was unresolved [18, 25] **(Fig 1B)**.

**Fig 4:**
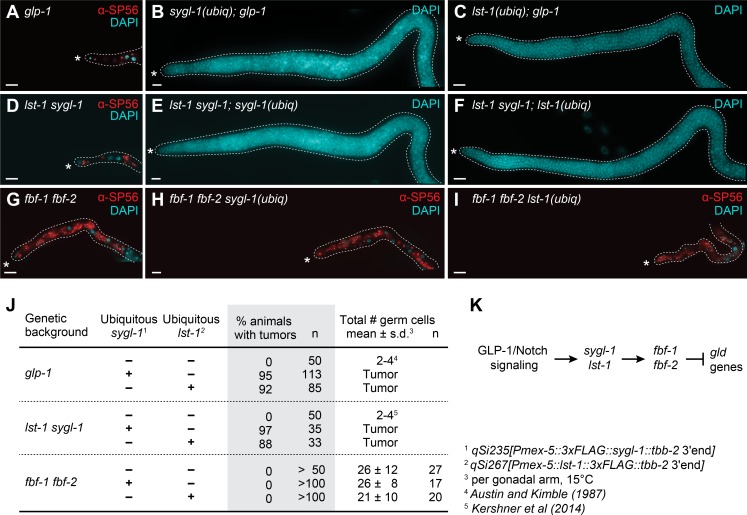
SYGL-1 and LST-1 tumor formation relies on FBF. (A-I) Epistasis tests using *sygl-1(ubiq)* or *lst-1(ubiq)* transgenes. All images are dissected young adult gonads stained with sperm marker SP56 (red) and DAPI (cyan). (A-C) Epistasis with *glp-1*. (A) GSC defect in *glp-1(q46)* null: the few GSCs in L1 larvae differentiate as sperm [14]. (B and C) Germline tumor in *sygl-1(ubiq); glp-1(q46)* null and *lst-1(ubiq); glp-1(q46)* null. (D-F) Epistasis with *lst-1 sygl-1*. (D) GSC defect in *lst-1(ok814) sygl-1(tm5040)* double mutant is indistinguishable from that of *glp-1* null [18]. (E and F) Germline tumor in *lst-1(ok814) sygl-1(tm5040); sygl-1(ubiq)* and in *lst-1(ok814) sygl-1(tm5040); lst-1(ubiq)*. (G-I) Epistasis test with *fbf-1 fbf-2*. GSC defect in *fbf-1(ok91) fbf-2(q704)* double mutant: GSCs made in larvae but not maintained past late L4 when all differentiate as sperm at 15°C and 20°C [15]. At 25°C, a small number of GSCs is maintained in adults [40]. (H and I) GSC defect similar to that of *fbf-1 fbf-2* double mutant in *fbf-1(ok91) fbf-2(q704) sygl-1(ubiq)* and *fbf-1(ok91) fbf-2(q704) lst-1(ubiq)*. See **S5 Fig** for confirmation that SYGL-1 and LST-1 are expressed and functional in these strains, and for characterization of these strains at 25°C. Conventions as in **Fig 1E–1J**; scale bar is 20 μm. In all strains, *sygl-1(ubiq)* is *qSi235[P*_*mex-5*_::*3xFLAG*::*sygl-1*::*tbb-2 3’end]* and *lst-1(ubiq)* is *qSi267[P*_*mex-5*_:: *lst-1*::*3xFLAG*::*tbb-2 3’end]*. (J) Summary of epistasis results. (K) Revised genetic model for GSC regulation. See text for further explanation.

We first asked whether *sygl-1(ubiq)* and *lst-1(ubiq)* can bypass GLP-1/Notch signaling. Whereas *glp-1(0)* mutants have no GSCs and make only a few sperm [14] (**Fig 4A**), *glp-1(0)* mutants develop germline tumors when either SYGL-1 or LST-1 is expressed ubiquitously (**Fig 4B and 4C**), confirming that *sygl-1* and *lst-1* function downstream of Notch signaling. We next asked if *sygl-1(ubiq)* and *lst-1(ubiq)* can drive germline tumors in double mutants lacking both *sygl-1* and *lst-1* endogenous loci. Whereas *lst-1 sygl-1* double mutants have no GSCs and only a few sperm (**Fig 4D**) [18], they become tumorous when either SYGL-1 or LST-1 is expressed ubiquitously (**Fig 4E and 4F**). Therefore, their tumor-forming activities are independent of each other, as expected.

Finally, we asked if *sygl-1(ubiq)* and *lst-1(ubiq)* can drive germline tumors in *fbf-1 fbf-*2 double mutants. Previous experiments relying on loss-of-function mutants suggested that *sygl-1* and *lst-1* might function at the same position as *fbf-1* and *fbf-2* in the genetic pathway [25] (**Fig 1B**). Here, using gain-of-function *sygl-1(ubiq)* and *lst-1(ubiq)*, we sought to clarify the relationship between *sygl-1*, *lst-1* and *fbf*. Because the GSC loss phenotype of *fbf-1 fbf-2* is the most severe at 15°C [15, 40] and *sygl-1(ubiq)* and *lst-1(ubiq)* are the most penetrant at 15°C (**S4A and S4B Fig**), our initial analysis focused on 15°C. At this temperature, *fbf-1 fbf-*2 adults cannot maintain GSCs (**Fig 4G**) [15]; remarkably, they also cannot maintain GSCs even when either SYGL-1 or LST-1 is expressed ubiquitously (**Fig 4H and 4I**). We confirmed that *sygl-1(ubiq)* and *lst-1(ubiq)* were expressed in *fbf-1 fbf-*2 mutants (**S5A–S5C Fig**) and that they made functional proteins (**S5D Fig**). Therefore, the *fbf-1 fbf-*2 GSC loss is epistatic to *sygl-1(ubiq)* and *lst-1(ubiq)* tumors, which we interpret as *sygl-1* and *lst-1* acting either upstream or in parallel to FBF. In other words, SYGL-1 and LST-1 require FBF to drive self-renewal at this temperature.

Although *sygl-1* and *lst-1* require FBF for tumor formation at 15°C, they unlikely drive stemness exclusively via FBF for two reasons: GSC loss is more severe in *lst-1 sygl-1* double mutants than in *fbf-1 fbf-2* double mutants [15, 18], and GSC loss in *fbf-1 fbf-2* double mutants can be enhanced by removal of either *lst-1* or *sygl-1* [25; this work]. In an attempt to see their FBF-independent function, we tested *fbf-1 fbf-2 sygl-1(ubiq)* and *fbf-1 fbf-2 lst-1(ubiq)* animals for tumor formation at 25°C, because at this temperature, the FBF requirement is relieved in that *fbf-1 fbf-2* mutants can maintain a small GSC pool [40]. Again at 25°C, both *sygl-1(ubiq)* and *lst-1(ubiq)* failed to generate germline tumors in the absence of FBF: *fbf-1 fbf-2 sygl-1(ubiq)* maintained a progenitor zone comparable in size to *fbf-1 fbf-2* double mutants while *fbf-1 fbf-2 lst-1(ubiq)* were more variable, with only 10% maintaining a progenitor zone and differentiation extending to the distal end in the other 90% (**S5E–S5J Fig).** Nonetheless, from lines of evidences noted above, SYGL-1 and LST-1 must have an FBF-independent role in stem cell maintenance.

In summary, GLP-1/Notch signaling from the niche is dispensable for SYGL-1 and LST-1 tumors, and SYGL-1 and LST-1 do not need each other for their activity (**Fig 4J**). In contrast, SYGL-1 and LST-1 rely on FBF to form tumors (**Figs 4J and S5E–S5J**). Therefore, our results are consistent with a genetic model in which *sygl-1* and *lst-1* act downstream of Notch but upstream or parallel to *fbf* (**Fig 4K**).

### SYGL-1 and LST-1 promote FBF activity rather than FBF expression

The reliance of SYGL-1 and LST-1 on FBF to promote tumor formation suggested two ideas for their molecular function. One possibility was that SYGL-1 and LST-1 regulate FBF expression. To test this notion, we compared FBF expression in germlines with and without SYGL-1 and LST-1, using a genetic background to circumvent the SYGL-1 and LST-1 requirement for GSC maintenance: *gld-2 gld-1* mutants make germline tumors independently of *sygl-1* and *lst-1* [18]. To detect FBF-1 and FBF-2, we used epitope-tagged transgenes, which are expressed and function biologically like their endogenous counterparts [27]. By staining, FBF-1 and FBF-2 proteins were expressed robustly both with and without SYGL-1 and LST-1 (**S6A–S6F Fig**), and Western blots confirmed the result (**S6G Fig**). We conclude that SYGL-1 and LST-1 are not required for FBF expression.

**Fig 5:**
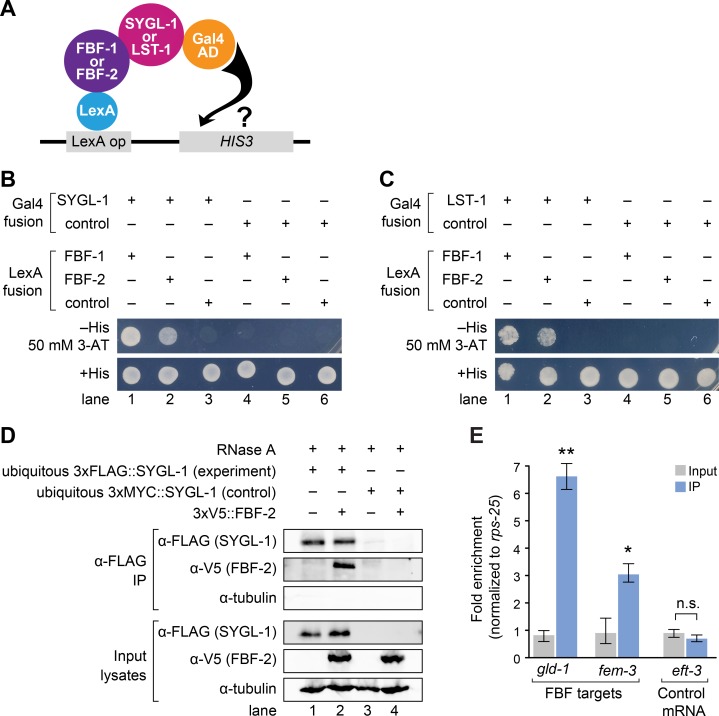
SYGL-1 and LST-1 interact physically with FBF. (A) Yeast two hybrid assay. Full length SYGL-1 or LST-1 was fused to Gal4 activation domain (AD); PUF repeats of FBF-1(121–614) or FBF-2(121–632) were fused to LexA binding domain (BD). Interaction activates transcription of *HIS3* gene. (B and C) Yeast growth assays tested interaction between SYGL-1 and FBF (B) or LST-1 and FBF (C). Yeast strains were monitored for growth on synthetic defined media (SD), either lacking histidine or with histidine as a control. A *HIS3* competitive inhibitor (3-AT) improved stringency. (D) SYGL-1 and FBF-2 co-immunoprecipitation (IP). Western blots probed with α-FLAG to detect SYGL-1, α-V5 to detect FBF-2, and anti-α-tubulin as a loading control. 2% of input lysates and 20% of IP elutes were loaded. Exposure times of input and IP lanes are different, so band intensities are not comparable. RNA degradation by RNase A was confirmed. Genotypes for each lane: (1) *sygl-1(tm5040); qSi235[P*_*mex-5*_::*3xFLAG*::*sygl-1*::*tbb-2 3’end];* (2) *sygl-1(tm5040); fbf-2(q931)[3xV5*::*fbf-2] qSi235[P*_*mex-5*_::*3xFLAG*::*sygl-1*::*tbb-2 3’end];* (3) *sygl-1(tm5040); qSi297[P*_*mex-5*_::*3xMYC*::*sygl-1*::*tbb-2 3’end];* (4) *sygl-1(tm5040); fbf-2(q932)[3xV5*::*fbf-2] qSi297[P*_*mex-5*_::*3xMYC*::*sygl-1*::*tbb-2 3’end]*. See **S7 Fig** for data supporting functionality of epitope-tagged FBF-2. (E) Quantitative PCR of two signature FBF target mRNAs and a control mRNA after α-FLAG IP, using either *3xFLAG*::*sygl-1(ubiq)* for the experiment or *3xMYC*::*sygl-1*(*ubiq*) as the control. Abundance of mRNAs in input (gray bars) and IPs (blue bars) was calculated with the ΔΔ C_T_ method, using *rps-25* for normalization. Error bar indicates standard error. Asterisks indicate a statistically significant difference by 1-way ANOVA with Tukey HSD *post hoc* test. * p<0.05, ** p<0.01.

An alternate idea posits that SYGL-1 and LST-1 act together with FBF, perhaps by enhancing FBF activity in a molecular complex. To ask if SYGL-1 and LST-1 physically interact with FBF, we first turned to the yeast two-hybrid assay (**Fig 5A**). Briefly, SYGL-1 or LST-1 was fused to the Gal4 activation domain (AD), and the PUF repeats of FBF-1 or FBF-2 were fused with the LexA DNA binding domain (BD). Binding was assayed by monitoring growth on minimal media lacking histidine, as a measurement of *HIS3* gene expression level. We imposed a stringent threshold by adding a competitive inhibitor of the *HIS3* enzyme (50 mM 3-AT) to minimize false positives. Robust growth was observed when either SYGL-1-AD or LST-1-AD was co-transformed with either FBF-1-BD or FBF-2-BD but not in controls (**Fig 5B and 5C**). We conclude that SYGL-1 and LST-1 both interact with FBF-1 and FBF-2 in yeast.

We next set out to ask if SYGL-1 and LST-1 might associate with FBF in nematodes. Immunoprecipitation of SYGL-1 and LST-1 from nematodes had been technically difficult because both proteins are normally expressed at low abundance and in only a subset of cells. To circumvent this problem, we attempted immunoprecipitation from *sygl-1(ubiq)* and *lst-1(ubiq)* tumorous animals. Immunoprecipitation was successful with SYGL-1 (**Fig 5D**), and subsequent biochemistry therefore focused on SYGL-1.

To ask if SYGL-1 associates with FBF in nematodes, we generated strains harboring a *sygl-1(ubiq)* transgene plus epitope-tagged 3xV5::FBF-2. Our experimental and control strains made germline tumors with 3xFLAG::SYGL-1 and 3xMYC::SYGL-1, respectively. The 3xV5::FBF-2 protein is functional and expressed (**S7A–S7D Fig**), as previously described [41]. We used FLAG antibodies to immunoprecipitate (IP) protein from both experimental and control strains; RNase A was added to all IPs to exclude RNA dependence of interactions. 3xFLAG::SYGL-1 co-immunoprecipitated with 3xV5::FBF-2 from the experimental but not the control strain, and this interaction was not dependent on RNA (**Fig 5D**). We conclude that SYGL-1 and FBF-2 associate with each other in nematodes and suggest that they form a complex.

FBF regulates many target mRNAs (see Introduction). If SYGL-1 works in a complex with FBF, then SYGL-1 protein might co-IP with FBF targets. To test this idea, we used the same strains described above and performed quantitative PCR of two established FBF targets, *gld-1* and *fem-3* mRNAs [15, 27, 42–44]. The experimental IP was enriched for both target mRNAs over the control IP, but it was not enriched for *eft-3* mRNA (**Fig 5E**), an mRNA not detected as a potential FBF target in genomic studies [27, 42]. We conclude that SYGL-1 associates specifically in nematodes with both FBF protein and with FBF target mRNAs.

### SYGL-1 and LST-1 repress *gld-1* expression post-transcriptionally

**Fig 6:**
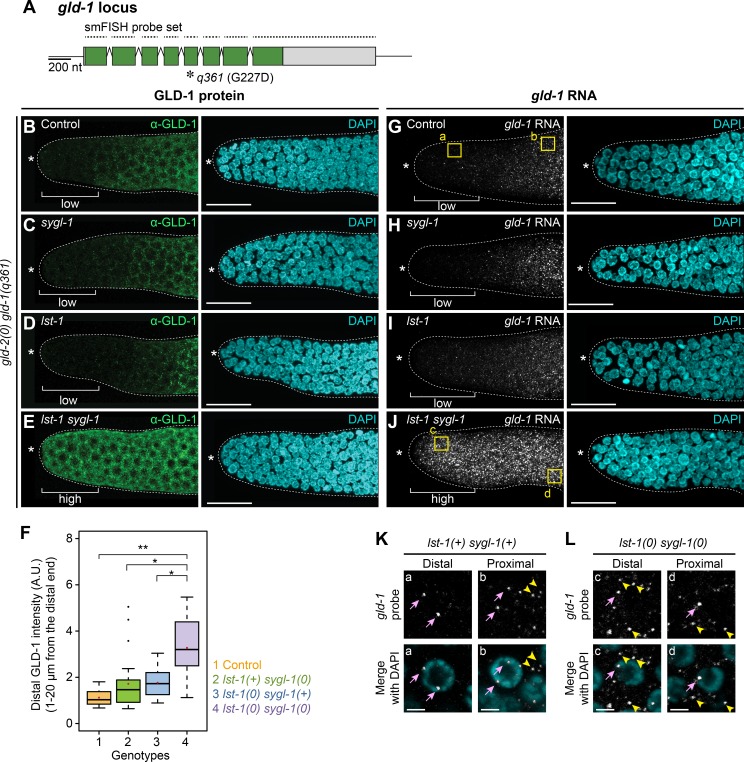
SYGL-1 and LST-1 repress *gld-1* expression post-transcriptionally in GSC pool. (A) Schematic of *gld-1(q361)*, a missense allele with a null phenotype [45] that generates mRNA and protein normally [30]. The smFISH probe set spanned the locus. See text for details. (B-E) GLD-1(q361) protein in distal gonads, stained with α-GLD-1 (green) and DAPI (cyan). Genotypes are: (B) *gld-2(q497) gld-1(q361)*; (C) *sygl-1(q828) gld-2(q497) gld-1(q361)*; (D) *lst-1(ok814) gld-2(q497) gld-1(q361)*; (E) *lst-1(ok814) sygl-1(q828) gld-2(q497) gld-1(q361)*. (F) Quantitation of GLD-1(q361). α-GLD-1 intensities in 0–20 μm (1-~5 gcd) from the distal end were averaged and plotted. Box plot conventions as in **Fig 2F**, genotypes as in **Fig 6B–6E**. Asterisks indicate a statistically significant difference by 1-way Welch’s ANOVA with Games Howell *post hoc* test. ** p<0.001, * p< 0.05. Number of gonads examined: Control, n = 23; *sygl-1*, n = 26; *lst-1*, n = 24; *lst-1 sygl-1*, n = 38. (G-J) *gld-1(q361)* transcripts in distal gonads, probed using smFISH (white) and DAPI (cyan). Genotypes as in **Fig 6B–6E**. All gonads (100%) had mRNA distributions as shown: control, n = 32; *sygl-1*, n = 35; *lst-1*, n = 41; *lst-1 sygl-1*, n = 38. (K and L) Pink arrows, nascent transcripts in nucleus. Yellow arrowheads, mature mRNAs in cytoplasm. Top, *gld-1* RNA; bottom, RNA merged with DAPI. (K) Magnifications from boxed areas in (G). In the presence of wild-type *sygl-1* and *lst-1*, distal germ cells possess nuclear transcripts, but little cytoplasmic mRNA, whereas proximal germ cells have both. (L) Magnifications from boxed areas in (J). Without *sygl-1* and *lst-1*, both distal and proximal germ cells contain nuclear and cytoplasmic *gld-1* transcripts. See **S8 Fig** for confirmation of *gld-1* probe specificity. All images are maximum intensity z-projections, except (K) and (L) show a single z-slice. Conventions as in **Fig 1E–1J**; scale bar is 20 μm in all images, except 2 μm in (K) and (L). n, number of gonadal arms.

The primary function of FBF in stem cell regulation is mRNA repression [16]. A crucial prediction of the idea that SYGL-1 and LST-1 work with FBF in a complex is that SYGL-1 and LST-1 should be required for repression of an FBF target mRNA. To test this idea, we examined *gld-1* mRNA, a well-established FBF target required for differentiation [15]. Previous experiments detected a subtle increase in GLD-1 expression in GSCs of *sygl-1* and *lst-1* single mutants [25]. To explore this further, we again used *gld-2 gld-1* mutants to remove both *sygl-1* and *lst-1* without changing cell fate. This time, however, we used *gld-1(q361)*, a missense mutant that abrogates GLD-1 protein function but produces detectable *gld-1* mRNA and GLD-1 protein [30, 45, 46] (**Fig 6A**). In this fashion, repression of *gld-1* mRNA was uncoupled from complications of GLD-1 function in the germline.

We first assayed expression of GLD-1(q361) protein. When either wild-type *sygl-1* or wild-type *lst-1* was present, GLD-1(q361) was expressed normally: barely detectable in distal-most germ cells and gradually increasing more proximally (**Fig 6B–6D**). However, when both *sygl-1* and *lst-1* were removed, GLD-1(q361) protein increased dramatically in the distal germline (**Fig 6E**), with quantitation revealing a three-fold increase on average (**Fig 6F**).

We next assayed expression of *gld-1(q361)* mRNA using single molecule fluorescence *in situ* hybridization (smFISH). Our probe was specific to *gld-1*: transcripts were patterned as described previously in wild type [41, 46] and cytoplasmic *gld-1* mRNAs were undetectable in *gld-1(q485)*, a deletion mutant that likely renders transcripts subject to non-sense mediated decay [45] (**S8 Fig**). Similar to the result with GLD-1 protein, *gld-1* mRNAs were barely detectable distally when either *sygl-1* or *lst-1* was present, but became easily detectable distally when both *sygl-1* and *lst-1* were removed (**Fig 6G–6J**). By contrast, nascent transcripts were seen in distal germ cell nuclei regardless of *sygl-1* and *lst-1* (**Fig 6K and 6L**). We conclude that SYGL-1 and LST-1 function post-transcriptionally to repress *gld-1* mRNA expression in the distal germline, a role that is strongly reminiscent of FBF activity. Collectively, our data support the idea that SYGL-1 and LST-1 partner with FBF to repress FBF target mRNAs in GSCs.

## Discussion

The *sygl-1* and *lst-1* genes are targets of niche signaling and crucial for GSC self-renewal [18]. Here we investigate the functions of SYGL-1 and LST-1 proteins, which had been a mystery. Our results support three major conclusions: SYGL-1 and LST-1 are sufficient for stem cell maintenance and can be oncogenic when unregulated; the spatial restriction of SYGL-1 and LST-1 proteins governs GSC pool size; and SYGL-1 and LST-1 work with FBF to restrict its RNA repression to stem cells. Our discussion places these results in context with implications for stem cell biology more broadly.

### SYGL-1 and LST-1 are sufficient for stem cell maintenance

We have found that ubiquitous expression of either SYGL-1 or LST-1 protein drives formation of extensive germline tumors, and that their tumor-forming activities do not require GLP-1/Notch signaling from the niche. The significance of this result is three-fold. First, SYGL-1 and LST-1 are not only required for GSC maintenance, albeit redundantly [18], but each on its own also drives stemness in the form of a tumor when ubiquitously expressed. This sufficiency underscores the importance of SYGL-1 and LST-1 as key stem cell regulators. Second, SYGL-1 and LST-1 are the primary targets of niche signaling for GSC maintenance: GLP-1/Notch signaling does not induce other regulators that must work with either SYGL-1 or LST-1 to maintain GSCs. Third, spatial restriction of SYGL-1 and LST-1 prevents tumor formation, making them prototypes for a new class of oncogenes.

Central to understanding the niche regulation of stem cells is the identification and characterization of key downstream effectors. Advances have been made in several model systems [e.g. 47–49], but examples of niche effectors with validated *in vivo* significance are rare. Perhaps the most striking parallels to SYGL-1 and LST-1 are *Ascl2* and *LgR5*, which encode niche signaling effectors in Wnt-regulated intestinal stem cells. Similar to SYGL-1 and LST-1, *Ascl2* and *LgR5* expression is limited to stem cells [50, 51], and ectopic expression promotes hyperplasia [52]. However, in stark contrast to SYGL-1 and LST-1, *Ascl2* and *LgR5* functions are not independent of niche signaling: *LgR5* enhances Wnt signaling and *Ascl2* works with Wnt-dependent transcription factors to induce a stem cell transcriptional signature [53]. Therefore, SYGL-1 and LST-1 stand out as direct targets of niche signaling that promote self-renewal by an intrinsic signaling-independent mechanism.

### Spatial restriction of SYGL-1 and LST-1 governs GSC pool size

**Fig 7:**
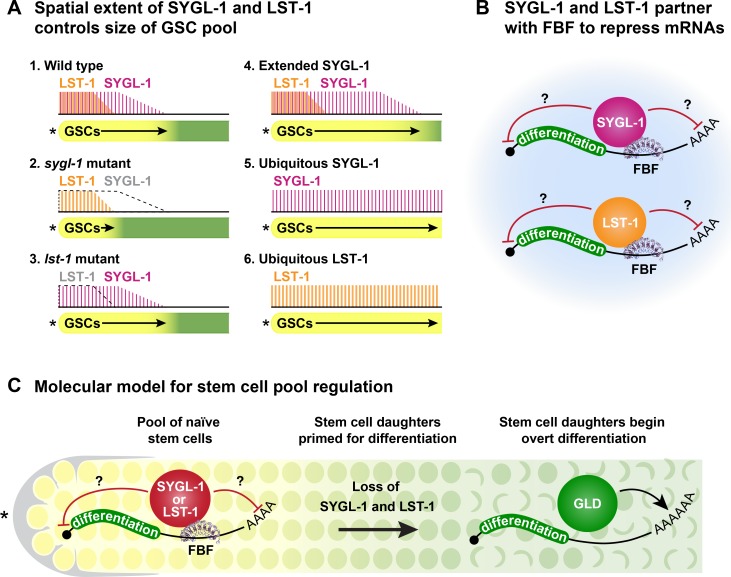
Models for stem cell pool regulation. (A) In each schematic, wild-type or manipulated extents of SYGL-1 (magenta) and LST-1 (orange) are shown above and GSC pool sizes are shown below. Wild type: GSC pool size corresponds to SYGL-1 rather than LST-1 extent; *sygl-1* mutant: pool size smaller than wild type and likely determined by smaller LST-1 extent; *lst-1* mutant: pool size not determined experimentally but likely similar to wild type, because progenitor zone is nearly the same size as normal; Extended SYGL-1 expression: moderate increase in SYGL-1 extent expands GSC pool (*tbb-2* 3’UTR transgene); Ubiquitous SYGL-1 expression: major expansion of SYGL-1 forms a massive tumor; Ubiquitous LST-1 expression: major expansion of LST-1 forms a massive tumor. (B) FBF forms a complex with SYGL-1 or LST-1 to repress differentiation RNAs. Red bars indicate repression; large pale blue circle represents an RNP granule. See text for explanation. (C) Loss of SYGL-1 and LST-1 triggers the switch from a naïve state to one primed-for-differentiation. See text for explanation.

Normally, SYGL-1 and LST-1 are spatially restricted to a region that correlates with estimates of the GSC pool (**Fig 7A**). We confirmed the biological significance of this spatial restriction in two ways. First, a moderate expansion of SYGL-1 expression led to a similar moderate expansion of pool size. Second, a major expansion of either SYGL-1 or LST-1 led to the formation of massive germline tumors. The simple conclusion is that the presence of either SYGL-1 or LST-1 promotes the stem cell fate, while their absence is critical for the transition towards differentiation. Logical corollaries are that spatial distributions of SYGL-1 and LST-1 govern the size of the GSC pool and that their loss facilitates the transition to a cell state primed for differentiation. A key question is how their spatial restriction is regulated. GLP-1/Notch signaling from the niche activates *sygl-1* and *lst-1* transcription in distal germ cells [18], but what regulates their disappearance? A partial answer is RNA regulation: the *sygl-1* 3’UTR restricts SYGL-1 protein expression compared to the *tbb-2* (tubulin) 3’UTR. In addition to RNA regulation, we suggest that SYGL-1 and LST-1 protein stabilities are also regulated. Despite the rapid kinetics of germ cell movement (~1 gcd per hour [54]), the distributions of *sygl-1* mRNA and protein are similar, as are those of *lst-1* mRNA and protein [19; this work]. Therefore, the SYGL-1 and LST-1 proteins must turn over as germ cells move proximally within the progenitor zone. Others have found that the proteolytic machinery is critical for progression from a stem cell state to a differentiated state in the progenitor zone [55, 56]. We suggest that SYGL-1 and LST-1 are likely targets of such proteolysis.

The *C*. *elegans* gonad therefore provides a new paradigm for how niche signaling can act through spatially restricted regulators to not only ensure the existence of stem cells but also to govern the size of a stem cell pool and facilitate the transition to a primed state. Spatial regulation is a common theme in animal development [57, 58] and extends to stem cell regulators. In addition to *Lgr5* and *Ascl2* (described above), the *Escargot*/Snail transcription factor follows a similar principle in intestinal stem cells in *Drosophila* and mouse models [59, 60]. More relevant to this work is the *Drosophila* PUF protein, Pumilio, which promotes GSC self-renewal [61, 62]. Pumilio is spatially restricted to GSCs and its ectopic expression generates germline tumors [63]. The clarifying advances of our work are an application of this theme to the maintenance of a stem cell pool, which is likely a broadly-used mechanism, and to a PUF protein partner rather than a PUF protein *per se* (see below).

### SYGL-1 and LST-1 partner with FBF to repress mRNA in stem cells

When this work began, the molecular functions of SYGL-1 and LST-1 were unknown (see Introduction). A first clue from this work was their cytoplasmic localization, which is consistent with a role in post-transcriptional regulation but can be explained in other ways. A more significant clue was that SYGL-1 and LST-1 cannot drive germline tumors in the absence of the FBF RNA-binding protein. One explanation might have been that SYGL-1 and LST-1 promote FBF expression, but that possibility was not confirmed: FBF-1 and FBF-2 were expressed in the absence of SYGL-1 and LST-1. An alternative idea was that SYGL-1 and LST-1 might work with FBF to promote mRNA repression. In support of that explanation, SYGL-1 and LST-1 interact with FBF-1 and FBF-2 in yeast two-hybrid assays; SYGL-1 co-immunoprecipitates from nematodes with both FBF-2 protein and with FBF target mRNAs; and SYGL-1 and LST-1 post-transcriptionally repress expression of one of those FBF targets in GSCs. These multiple lines of evidence support the model that SYGL-1 and LST-1 partner with FBF to repress mRNAs in GSCs (**Fig 7B)**. We emphasize that SYGL-1 and LST-1 must also have FBF-independent functions, because the *lst-1 sygl-1* phenotype is more severe than the *fbf-1 fbf-2* phenotype [15, 18], and because single *sygl-1* and *lst-1* mutants enhance the *fbf-1 fbf-2* phenotype [25; this work]. The *fog-1* gene, which encodes a cytoplasmic polyadenylation element binding (CPEB) related protein [64, 65], redundantly promotes GSC self-renewal with FBF in that *fog-1 fbf-1 fbf-2* triple mutants contain a GSC loss similar to that of *glp-1* null [66]. We speculate that the FBF-independent functions of SYGL-1 and LST-1 may involve regulation of FOG-1 protein or key FOG-1 mRNA targets. But of course, other possibilities exist. Regardless, this work shows conclusively that SYGL-1 and LST-1 have an FBF-dependent function and that they likely operate with FBF in a complex.

SYGL-1 and LST-1 stand out among PUF partners as the first to be essential for GSC maintenance, the first to be spatially restricted to the stem cell region, the first to affect size of a stem cell pool, the first to be tumorigenic when overexpressed, and the first to be essential for mRNA repression in GSCs. Previously identified FBF partners include NOS-3, a Nanos homolog which is expressed throughout the germline [67, 68], and CPEB/CPB-1, which is expressed and functions in spermatocytes [64, 69]. Two other FBF partners, GLD-2 and GLD-3, activate mRNAs and promote germ cell differentiation [70–72], a function opposite that of SYGL-1 and LST-1. The molecular mechanisms by which SYGL-1 and LST-1 repress RNAs await future studies. The simplest possibility is that they enhance FBF recruitment of the Not1 deadenylase complex, a conserved mode of PUF repression from yeast to humans [73–75]. Another idea is that SYGL-1 or LST-1 influences the sequence specificity and kinetics of FBF binding to target mRNAs, analogous to reports for other PUF partners such as CPB-1 for FBF [76] and Nanos or Brat for *Drosophila* Pumilio [77, 78]. A third thought is that SYGL-1 and LST-1 repress RNAs by recruiting them to sites of repression in RNP granules. The emerging view of low complexity proteins as RNA granule scaffolds [e.g. 79, 80] coupled with the punctate or granular appearance of SYGL-1 and LST-1 make this third possibility attractive, but it remains speculative. Given that several mechanisms remain plausible, we note that SYGL-1 and LST-1 may employ distinct biochemical mechanisms, despite their biological redundancy in GSC maintenance and their molecular redundancy in *gld-1* mRNA repression. A tantalizing future direction is to ask if similar counterparts of SYGL-1 or LST-1 exist in other vertebrate stem cell models to enhance the repressive activity of PUF proteins, Pum1 and Pum2.

### Molecular model for governing the naïve state and size of a stem cell pool

Our findings together with previous studies support a model for how niche signaling is coordinated with intrinsic stem cell regulators to establish a GSC pool with stem cells in their naïve state and then facilitate the transition to a state primed for differentiation (**Fig 7C**). Essentially, Notch signaling localizes the GSC pool by activating expression of key intrinsic stem cell regulators, SYGL-1 and LST-1, which partner with FBF to repress differentiation mRNAs and thereby promote the naïve state (**Fig 7C, left)** [14, 15, 18, 19; this work]. Pool size is established roughly by Notch signaling, which activates *sygl-1* transcription in a steep gradient across the pool [18, 19]. However, *sygl-1* mRNAs are less graded and therefore transform the steep transcriptional gradient into a markedly less steep RNA gradient [19]. Here, we show that SYGL-1 protein abundance disappears in a pattern closely mirroring loss of its mRNAs. We propose that removal of these key FBF partners drives the transition from a naïve to a primed state (**Fig 7C, middle)**, and that loss of SYGL-1 and LST-1 triggers entry into a primed state by releasing *gld-1* and likely other RNAs from repression (**Fig 7C, right**). We note that FBF is present not only in the GSC pool but also in primed cells and cells beginning overt differentiation (entry into meiotic prophase) [15, 41, 81]. However, repression of FBF target mRNAs occurs in the distal germline [15, 40, 75, 82–84] and is strongest in the distal-most region or the naïve GSC pool [11]. This pattern suggests that FBF in primed cells is becoming less repressive as SYGL-1 and LST-1 are lost; indeed, FBF may be transitioning to an activating mode in this primed region [10, 75]. Two other FBF partners, GLD-2 and GLD-3, activate FBF-bound RNAs [75], suggesting the possibility of a partner exchange during the transition in primed cells. One can imagine that SYGL-1 and LST-1 might be displaced by competition of other FBF partners or they might be removed by spatially regulated proteolysis. Although our model is surely oversimplified, it provides a heuristic framework for future explorations of stem cell pool regulation. For example, the model poises our thinking for analysis of both the mechanism and kinetics of transition from a naïve state to a primed state, which are likely to have profound consequences on pool regulation. Regardless, this model provides critical insights into how niche signaling is coordinated with downstream intrinsic effectors to govern the existence of a stem cell pool and its size.

## Material and methods

### Nematode strains and maintenance

Most strains were maintained and characterized at 20°C under standard conditions [85], except as follows: strains containing *emb-30(tn377ts)* were maintained at 15°C; strains harboring *sygl-1(ubiq)* tumor transgenes (*qSi235*, *qSi297*) were maintained on *sygl-1*(RNAi) feeding bacteria, and strains with *lst-1(ubiq)* tumor transgenes (*qSi267*) were maintained on *lst-1*(RNAi) (see RNAi section of Methods). The wild type was N2 Bristol strain. Alleles are as follows: LG*I*: *gld-2(q497*) [86], *gld-1(q485)* [33], *gld-1(q361)* [45], *fog-3(q520)* [34], *lst-1(ok814)* [87], *lst-1(q826)* (this work), *sygl-1(tm5040)* [18]. LG*II*: *fbf-2(q704*) [15], *fbf-2(q738)* [81], *fbf-1(ok91)* [15]. LG*III*: *glp-1(q46)* [14], *emb-30(tn377ts)* [29], *unc-119(ed3)* [88]. Balancers are as follows: LG*I*: *hT2[qIs48]* [89], LG*II*: *mIn1[mIs14 dpy-10(e128)]* [90], LG*III*: *hT2[qIs48]* [89]. Transgenes are as follows: LG*II*: *weSi2[P*_*mex-5*_::*GFP*::*his-58*::*tbb-2 3’end*, *unc-119 (+)]* [91], *qSi22[P*_*lst-1*_::*lst-1*::*1xHA*::*lst-1 3’end*, *unc-119 (+)]* (this work), *qSi49[P*_*sygl-1*_::*3xFLAG*::*sygl-1*::*sygl-1 3’end*, *unc-119(+)]* (this work), *qSi69[P*_*lst-1*_::*lst-1*::*3xFLAG*::*lst-1 3’end*, *unc-119 (+)]* (this work), *qSi75[P*_*fbf-2*_::*3xFLAG*::*fbf-2*::*fbf-2 3’end*, *unc-119(+)]* [27], *qSi150[P*_*sygl-1*_::*3xFLAG*::*sygl-1*::*tbb-2 3’end*, *unc-119(+)]* (this work), *qSi232[P*_*fbf-1*_::*3xFLAG*::*fbf-1*::*fbf-1 3’end*, *unc-119(+)]* [27], *qSi235[P*_*mex-5*_::*3xFLAG*::*sygl-1*::*tbb-2 3’end*, *unc-119(+)]* (this work), *qSi267[P*_*mex-5*_::*lst-1*::*3xFLAG*::*tbb-2 3’end*, *unc-119(+)]* (this work), *qSi297[P*_*mex-5*_::*3xMYC*::*sygl-1*::*tbb-2 3’end*, *unc-119(+)]* (this work). LG*IV*: *qSi93[P*_*lst-1*_::*lst-1*::*1xHA*::*lst-1 3'end*, *unc-119 (+)]* (this work). Alleles generated using CRISPR-Cas9 are as follows: LG*I*: *lst-1(q1004)[lst-1*::*3xV5]* (this work), *lst-1(q1008)[lst-1*:: *3xOLLAS]* (this work), *sygl-1(q828)* (this work), *sygl-1(q964)[3xMYC*::*sygl-1]* (this work), *sygl-1(q983)[3xOLLAS*::*sygl-1]* (this work), *sygl-1(q1015)[sygl-1*::*1xV5]* (this work). LG*II*: *fbf-2(q931)[3xV5*::*fbf-2]* (this work), *fbf-2(q932)[3xV5*::*fbf-2]* (this work). A complete list of strains used in this study is summarized in **S1 Table**.

### Generation of *C*. *elegans* alleles and transgenes

Single-copy transgenes were generated using the Mos-1 mediated single-copy insertion method (MosSCI) [20–22]. Briefly, plasmids containing the gene of interest were constructed using the Gibson assembly method [92] and microinjected at 50 ng/μl along with transposase and co-injection markers to target the *ttTi5605* or *cxTi10816* sites. Several transgenes were generated and maintained on RNAi feeding bacteria. Those requiring *sygl-1*(RNAi) were *qSi235[*_*Pmex-5*_::*3xFLAG*::*sygl-1*::*tbb-2 3’end*, *unc-119(+)]* and *qSi297[P*_*mex-5*_::*3xMYC*::*sygl-1*::*tbb-2 3’end*, *unc-119(+)]*. That requiring *lst-1(RNAi)* was *qSi267[P*_*mex-5*_::*lst-1*::*3xFLAG*::*tbb-2 3’end*, *unc-119(+)]*. At least two independent lines for each construct were analyzed, and results of one representative line are reported. A complete list of generated alleles and plasmids used to generate MosSCI transgenes can be found in **S2 Table** and **S4 Table** respectively.

*sygl-1(q828)* was generated using CRISPR/Cas9 gene editing [93]. Briefly, three 25 ng/μl *sygl-1* sgRNAs, a 50 ng/μl repair template designed to substitute the *sygl-1* coding region with *Caenorhabditis briggsae unc-119*, and 50 ng/μl pDD162 encoding Cas-9 were microinjected into the *unc-119(ed3)* strain with co-injection markers, and progeny were screened for the Unc movement rescue. The substitution of the *sygl-1* gene with the *unc-119* gene resulted in deletion of the *sygl-1* coding region and was verified by sequencing.

The alleles *fbf-2(q931)*, *fbf-2(q932)*, *sygl-1(q964)*, *sygl-1(q983)*, *lst-1(q1004)*, *lst-1(q1008)*, and *sygl-1(q101*5*)* were generated by RNA protein complex (RNP) CRISPR [23, 24]. Briefly, injection mix containing gene-specific crRNAs (10 μM, IDT Alt-R^TM^), *dpy-10* or *unc-58* co-CRISPR crRNAs (4 μM, IDT Alt-R^TM^), tracrRNAs (14 μM, IDT Alt-R^TM^), gene-specific repair oligo (4 μM) or repair plasmid (50 ng/μl), *dpy-10* or *unc-58* co-CRISPR repair oligo (1.4 μM), and Cas-9 protein (25 μM) was prepared. Strains were microinjected and the progeny were screened using PCR for edits. All CRISPR alleles were verified by sequencing and outcrossed 2–4 times with wild type prior to analysis. A complete list of reagents used to generate CRISPR alleles can be found in **S3–S5 Tables**.

To obtain *lst-1(q826)*, a *sygl-1* enhancer screen was performed with EMS mutagenesis as described [85], with minor modifications. Briefly, *sygl-1(tm5040)* hermaphrodites of the fourth larval stage (L4) were mutagenized with 25 mM Ethyl methanesulfonate (Sigma #M0880) for 4 hours at room temperature. F1 progeny were singled and maintained at 15°C, and F2 self-progeny were screened for germline proliferation defective (Glp) [14] mutants. Details of this mutagenesis screen are available upon request. The *lst-1* locus was sequenced from DNA extracted from Glp animals to identify the *lst-1(q826)* allele, which was outcrossed 10 times with wild type prior to analysis.

### Reannotation of *sygl-1* and *lst-1* gene structures

The *sygl-1* and *lst-1* gene structures reported here are based on 5’ rapid amplification of cDNA ends (RACE), genome-wide mRNA sequencing data (WormBase release 255), and ribosome profiling data [94]. Specifically, the *sygl-1* 5’UTR, the *lst-1* 5’UTR, and the *lst-1* start codon have been re-annotated. Most importantly, our reported *lst-1* start codon removes 70 amino acids from the previously mis-annotated versions [18] and is consistent with evolutionary data from *C*. *briggsae*.

For 5’ RACE, total RNA was extracted from young adult wild type (24 hours after L4 at 20°C) using TRIzol (Invitrogen #15596026). 1 μg of total RNA was converted to cDNA with SuperScript III (Invitrogen #18080051) using *sygl-1_RT_primer* (5’-AGCGACGAGTTGAAGAGACTC-3’) or *lst_RT_primer* (5’-GGTGCGACATGTCTCGTGGATC-3’). cDNAs were purified (QIAquick PCR purification kit, Qiagen #28106), tailed with cytosines using Terminal Deoxynucleotidyl Transferase (Invitrogen #EP0161), and then PCR amplified using the following primers: for *sygl-1*, primary PCR used *Anchor_Primer* (5’-GGCCACGCGTCGACTAGTACGGGIIGGGIIGGGIIG-3’) with *sygl-1_primary* (5’-TCGACGAGCGAGTCAGTCTC-3’); secondary PCR used *Universal_amplification_primer* (5’-GGCCACGCGTCGACTAGTAC-3’) with *sygl-1_secondary* (5’-CGCCTCCGGTTGACGATGATG-3’); and tertiary PCR used *Universal_amplification_primer* with *sygl-1_tertiary* (5’-AGACGATGAGGTGGACATG-3’). An additional tertiary reaction was carried out to improve the signal to noise ratio. For *lst-1*, primary PCR used *Anchor_Primer* (5’-GGCCACGCGTCGACTAGTACGGGIIGGGIIGGGIIG-3’) with *lst_primary* (5’-GAGTTGAAGCAGTTGCTTCGG-3’) and secondary PCR used *Universal_amplification_primer* (5’-GGCCACGCGTCGACTAGTAC-3’) with *lst_secondary* (5’-gtgttgcgacttcgagtagg-3’). All amplified products were analyzed by Sanger sequencing.

### Phenotype analyses: Brood counts, sterility and embryonic lethality

L4 hermaphrodites were placed onto individual plates at 20°C. At 6 to 12 hour intervals, the hermaphrodite was moved to a new plate and the embryos were counted for sterility and brood counts. Several days later, hatched progeny on each plate were counted to determine embryonic lethality.

### Assessment of progenitor zone size

All characterization of progenitor zone (PZ) size was done in animals raised at 20°C until 24 hours after L4, except in **S5J Fig** where animals were raised at 25°C until 16–18 hrs after L4. To visualize nuclear morphology, gonads were dissected, fixed, and stained with DAPI (see immunostaining and DAPI staining section below for dissection and fixation methods). To determine the PZ size, gonads were imaged using a confocal microscope with a z-stack depth of 0.4–0.5 μm. Next, the boundary between PZ and Transition Zone (TZ) was determined by conventional criteria [95]. Briefly, many germ cells in the TZ have entered meiotic prophase and hence have a crescent-shaped nuclear morphology. The PZ/TZ boundary was scored as the distal-most cell row with at least two crescent-shaped nuclei. Finally, the cells within the progenitor zone were counted manually using the cell-counter plug-in in FIJI/Image J, with each DAPI-stained nucleus scored as a single cell.

### Germ cell number estimation in *fbf* gonads

To estimate the number of germ cells in *fbf-1 fbf-2* gonads reported in **S5D Fig**, compact nuclei typical of mature sperm in a gonadal arm were counted manually using the cell counter tab in Openlab 5.5.2 (PerkinElmer). Next, the number of sperm was converted to the number of germ cells (four sperm are made from one germ cell).

### Estimation of SYGL-1 or LST-1 positive germ cells

To estimate the number of distal germ cells expressing SYGL-1 or LST-1, JK4996, JK5073, JK5205, JK5263, JK5893, JK5929 and JK6002 were raised at 20°C until adulthood (24 hours after L4), along with appropriate wild-type controls. Gonads were dissected, fixed, and stained with anti-FLAG, anti-OLLAS, anti-V5, or anti-HA (see immunostaining section below) and imaged using the confocal microscope. Next, the number of distal germ cells that contained positive V5 or OLLAS signal (SYGL-1) or positive HA, FLAG, or V5 signal (LST-1) above the background level was manually scored, using the cell-counter plugin in FIJI/Image J.

### *emb-30* assay

The assay was performed as described [11] with minor modifications. DG627, JK5233, JK5235 animals were raised at 15°C until 36 hours past mid-L4, then moved to plates pre-incubated at 25°C and maintained at 25°C for 12.5 hours. We chose 12.5 hours because germ cell counts became unreliable with longer times (nuclear morphology became increasingly compromised after incubations of 13 hours and longer). Next, gonads were dissected, fixed, and stained for anti-PH3, anti-GLD-1 and DAPI (see staining section below). To estimate the number of cells within the distal pool, we manually counted the number of M-phase arrested germ cells distal to the GLD-1 boundary (as assessed by DAPI morphology and PH3 staining) using the cell counter tab in Openlab 5.5.2 (PerkinElmer). Scoring was done blind to genotype. We excluded samples with abnormal, fragmented nuclei that made cell counting unreliable (22–49% per genotype). We note that not every nucleus distal to the GLD-1 boundary was arrested in M-phase in some gonads but these few nuclei were included in the “distal pool” counts.

### RNAi

Feeding RNAi was performed as described [96] using *sygl-1* or *lst-1* clones from the Ahringer RNAi library [97]. Bacteria were grown overnight at 37°C in 2xYT media containing 25 μg/μl carbenicillin and 50 μg/μl tetracycline. Cultures were concentrated, seeded onto Nematode Growth Medium (NGM) plates containing 1mM IPTG, then induced overnight before plating worms.

### *sygl-1(ubiq)* and *lst-1(ubiq)* germline tumor assays

To induce *sygl-1(ubiq)* and *lst-1(ubiq)* germline tumors, L4 P0 animals were transferred from RNAi bacteria to OP50-seeded NGM plates, and subsequent generations were monitored using a dissecting scope for germline tumor formation. In some experiments, gravid adults were bleached between generations to synchronize populations. All experiments with *sygl-1(ubiq)* and *lst-1(ubiq)* were carried out at 15°C to maximize tumor penetrance, except those in **S4A and S4B Fig**, where tumor penetrance was tested with different temperature regimens, and in **S5E–S5J Fig**, where epistasis with *fbf-1 fbf-2* was assayed at 25°C. For most *sygl-1(ubiq)* tumors, data were obtained in the F3 generation after removal from RNAi, and for most *lst-1(ubiq)* tumors, data were obtained in F2 after removal from RNAi. Two exceptions were: (1) For epistasis experiments requiring a balancer for strain maintenance (JK5401, JK5403, JK5538, JK5585; see **Fig 4B**, **4C**, **4E**, **4F**), tumors were scored in F1, because all F1 balancer-carrying animals were tumorous so additional generations could not be obtained. (2) For 25°C epistasis experiments with *fbf-1 fbf-2* (see **S5E–S5J Fig**), we scored in F8 (*sygl-1*) and F7 (*lst-1*) after removal from RNAi to maximize tumor penetrance.

### Immunostaining and DAPI staining

Staining followed established protocols [98] with minor modifications. Briefly, staged animals were dissected in PBStw (PBS + 0.1% (v/v) Tween-20) with 0.25 mM levamisole to extrude gonads. Tissues were fixed in 2% (w/v) paraformaldehyde diluted in 100 mM K_2_HPO_4_ (pH 7.2) for 10 minutes when using anti-FBF-1 and anti-PGL-1 antibodies. For all other antibodies, tissues were fixed in 3%(w/v) paraformaldehyde diluted in 100 mM K_2_HPO_4_ (pH 7.2) for 30 minutes. Post fixation, all samples were permeabilized with ice-cold methanol or PBStw + 0.2% (v/v) Triton-X for 5–10 minutes. Next, they were blocked with either 30% (v/v) goat serum diluted in PBStw (for anti-FLAG) or 0.5% (w/v) bovine serum albumin diluted in PBStw (all other antibodies) for 1 hour. For primary antibodies, samples were incubated overnight at the following dilutions in the blocking solution: Mouse anti-FLAG (1:1000, M2 clone, Sigma #F3165), Rabbit anti-GLD-1 (1:100, Gift from E. Goodwin), Rat anti-HA (1:100, 3F10 clone, Roche #11867423001), Rabbit anti-REC-8 (1:100, [30]), Rat anti-FBF-1 (1:5, [15]), Mouse anti-SP56 (1:200, [99]), Mouse anti-PH3 (1:200, Cell Signaling #9706), Rabbit anti-PGL-1 (1:100 [32]), Mouse anti-V5 (1:1000, Bio-Rad #MCA1360), Rat anti-OLLAS (1:2000, L2 clone, Novus Biologicals #NBP1-96713). For secondary antibodies, samples were incubated for 1 hour at room temperature at the following dilutions: Donkey Alexa 555 anti-mouse (1:1000, Invitrogen #A31570), Goat Alexa 555 anti-rabbit (1:1000, Invitrogen #A21429), Goat Alexa 488 anti-rabbit (1:1000, Invitrogen #A11034), Donkey Alexa 488 anti-rat (1:500, Invitrogen #A21208), Donkey Alexa 647 anti-mouse (1:500, Invitrogen #A31571). To visualize DNA, DAPI was included at a final concentration of 0.5–1 ng/μl during the last 10 minutes of secondary antibody incubation. Vectashield (Vector Laboratories #H-1000) was used as mounting medium.

### *In situ* hybridization

Single molecule FISH (smFISH) was performed as described [19, 41, 100]. Custom Stellaris FISH probes were designed by utilizing the Stellaris FISH probe designer (Biosearch Technologies, Inc) available online at www.biosearch.com/stellarisdesigner. The *gld-1* probe set contains 48 unique probes labeled with CAL Fluor Red 610 and was used at a final concentration of 0.25 μM.

### Microscopy

For the compound microscopy data shown in **Fig 4,** images were taken using a Zeiss Axioskop with Hamamatsu CCD or ORCA cMOS camera equipped with 63x 1.4NA Plan Apochromat oil immersion objective. Carl Zeiss filter sets 49, 38, and 43HE were used for the visualization of DAPI, Alexa 488, and Alexa 555 respectively. An X-Cite 120Q lamp (Lumen Dynamics) was used as the fluorescence light source. Openlab 5.5.2 (PerkinElmer) and Micromanager [101, 102] were used as acquisition software. For all other figures, a Leica TCS SP8 confocal microscope driven by LAS software version 3.3.1 or X was used. This laser scanning confocal microscope was equipped with Photomultiplier (PMT) and Hybrid detectors (HyD). For all images, a 63x 1.4NA HC Plan Apochromat oil immersion objective was used with 100–200% zoom for immunostaining, and 300% zoom for single molecule FISH, using the standard scanner with 400Hz scanning speed. For figure preparation, contrast was linearly adjusted in Adobe Photoshop identically across all samples. In some cases, images were merged using the stitching plugin in FIJI/Image J [103] to generate whole gonad images.

### Fluorescence quantitation

All images used for quantitation were acquired using the sequential scan mode on the Leica TCS SP8, under the same conditions across all samples. Next, average intensity of multiple z-slices was projected onto a single plane. To eliminate signal intensities outside of the gonad (i.e. intestine), a separate binary mask was created by thresholding Nomarski images of the gonad taken at the same time; the binary mask was then multiplied to other channels such that only signals within the gonad would be considered for quantitation. Next, intensity at a given distance “x” from the distal tip of the gonad was averaged over five-micron intervals (“moving average”). For simplicity, distance from the distal end was converted to conventional germ cell diameters, using a conversion ratio of 4.55 μm for one germ cell diameter [19]. A custom MATLAB script was used to process steps described above.

### Yeast two hybrid

Modified yeast two-hybrid assays were performed as described [104]. Briefly, *sygl-1* cDNA encoding full-length SYGL-1 (a.a. 1–206) or *lst-1* cDNA encoding full-length LST-1 (a.a. 1–328) was cloned into the *Nco* I and *Xho* I sites in pACT2 (Gal4 activation domain plasmid) to generate pJK1580 and pJK2015, respectively. Regions encoding FBF-1 (a.a. 121–614) and FBF-2 (a.a. 121–632) were cloned into the *Eco*R I and *Sal* I sites in pBTm116 (LexA binding domain plasmid) to generate pJK2019 [67] and pJK2017, respectively. Plasmids were co-transformed into a L40-*ura* strain using the Te-LiAc method [105]. *His3* reporter activity was assayed on synthetic defined medium (SD) supplemented with –Leu–Trp–His containing 50 mM 3-Amino-1,2,4-triazole (Sigma #A8056), or –Leu–Trp plates as controls for 4 days at 30°C.

### Immunoprecipitations and Western blots

JK5366, JK5574, JK5783, and JK5844 animals were raised at 15°C until they developed germline tumors as young adults (12 hours after L4) (see tumor assay above). Animals were washed twice with M9 buffer [3 g/L KH2PO4, 6 g/L NaHPO4, 5 g/L NaCl, and 1 mM MgSO4] and cross-linked with 1% (w/v) formaldehyde for 10 minutes at room temperature (RT). Pellets were resuspended in 1 ml lysis buffer [50 mM HEPES pH 7.5, 150 mM NaCl, 1 mM EDTA, 1% (v/v) Triton-X, complete Protease inhibitor cocktail (Roche)], frozen in liquid nitrogen, and pulverized with mortar and pestle for 10 minutes. Lysates were cleared twice by centrifugation (12,000g, 10 minutes), and the total protein concentration was measured by Direct Detect Spectrophotometer (Millipore). To prepare antibody conjugated beads, 30 μg anti-FLAG (M2 clone, Sigma #F3165) was incubated with 4.5 mg protein G Dynabeads (Novex, Life Technologies, #10003D) for 30 minutes at RT. Next, 20 mg lysates were incubated with the antibody-bead mixture for 4 hours at 4°C, with the presence of RNase A at 10 μg/ml. RNA degradation was confirmed by isolating total RNA from post-IP lysates using TRIzol LS (Invitrogen #10296028) and analyzing on agarose gels. Beads were pelleted, washed four times with lysis buffer, and then two times with wash buffer [50 mM HEPES pH 7.5, 0.5 M NaCl, 1 mM EDTA, 1% (v/v) Triton X-100]. Samples then were eluted with elution buffer [1% (w/v) SDS, 250 mM NaCl, 1 mM EDTA, 10 mM TRIS pH 8] for 10 minutes at 65°C, and analyzed using SDS-PAGE on an 8% or 12% acrylamide gel.

To probe FBF abundance in **S6G Fig**, N2, JK5181, JK5182, JK5600, JK5602, JK5603, and JK5604 animals were raised at 20°C to young adulthood (12 hours after L4 stage). 40 animals were boiled in 2x Laemmli buffer and then analyzed by SDS-PAGE on a 4–20% gradient gel (Lonza #58527).

For primary antibodies, blots were incubated overnight at 4°C at the following dilutions: Mouse anti-FLAG (1:1000, M2 clone, Sigma #F3165), Mouse anti-V5 (1:1000, Bio-Rad #MCA1360), Mouse anti-actin (1:40,000, C4 clone, Millipore #MAB1501), Mouse anti-α-tubulin (1:20,000, Sigma #T5168). For secondary antibody, blots were incubated for 1 hour at RT with Donkey HRP-conjugated anti-mouse (1:10,000, Jackson ImmunoResearch). Immunoblots were developed using SuperSignal^TM^ West Pico/Femto Sensitivity substrate (Thermo Scientific #34080, #34095) and imaged using an ImageQuant LAS4000 (GE Healthcare). FIJI/Image J was used to calculate blot intensity. For final figure preparations, contrast of the blot was linearly adjusted in Adobe Photoshop.

### RNA immunoprecipitation (IP) and quantitative PCR

JK5366 and JK5574 were raised at 15°C until they developed germline tumors as young adults (see tumor assay above). Immunoprecipitation was done as above except that formaldehyde cross-linking and RNase treatment of lysates were omitted. Instead, lysis buffer contained 1 U/μl SUPERase·In RNase inhibitor (Ambion #AM2694). Successful IP was confirmed by analyzing 10% of elution by Western blot, and RNA was eluted from the rest of the beads with 0.5 ml TRIzol (Invitrogen #15596026). RNA was purified by RNeasy Micro kit (Qiagen #74004) including DNase I treatment on column. Purified RNA was checked for integrity, and converted to cDNA with Superscript III first strand synthesis kit (Invitrogen #18080051) using random-hexamers as primers. Quantitative PCR was carried out using a Roche Lightcycler 480 with TaqMan gene expression assays (Applied Biosystems). Enrichment was calculated by ΔΔ C_T_ method [106]. Taqman probes used are as follows: *gld-1*, Ce02409901_g1; *eft-3*, Ce02448437_gH; *rps-25*, Ce02464216_g1; *fem-3*, Ce02457444_g1.

### Statistical analysis

Statistical tests are indicated in figure legends with sample sizes. In most cases, one-way ANOVA and *post-hoc* Tukey multiple comparison tests were performed to calculate p-values. In cases where equal variance assumption of ANOVA was not established at p<0.01 (Levine’s test), Welch’s one-way ANOVA (modified ANOVA with heteroskedastic data) and *post-hoc* Games-Howell multiple comparison tests were performed to calculate p-values. All statistics were performed in R.

## Supporting information

S1 Fig Characterization of *sygl-1* and *lst-1* epitope-tagged alleles. (A-C) SYGL-1 and LST-1 in dissected gonads. Representative z-projection images of staining with α-V5, using *sygl-1*::*1xV5* and *lst-1*::*3xV5* epitope tagged alleles. Conventions are as in **Fig 1E–1J**; scale bar is 20 μm. Genotypes are (A) *sygl-1(q1015)[sygl-1*::*1xV5]*, (B) *lst-1(q1004)[lst-1*::*3xV5]*, (C) wild type. In addition to distal expression within the progenitor zone (PZ), SYGL-1 and LST-1 are present in the proximal gonad, consistent with their mRNA expression [18, 19]. (D and E) Functionality of epitope-tagged SYGL-1 or LST-1 transgenic proteins (D) or endogenous alleles (E). Because *lst-1 sygl-1* double mutants are 100% sterile but single mutants are fertile [18], functionality of epitope-tagged transgenes or endogenous alleles was tested by scoring fertility in the appropriate mutant background.
(TIF)

S2 Fig Characterization of *sygl-1* and *lst-1* single mutants. (A and B) Progenitor zone (PZ) size in *sygl-1* and *lst-1* mutants. (A) PZ length measured in number of germ cell diameters (gcd) from distal end. The averages and standard deviations are as follows: wild type, 19 ± 2 (n = 13); *sygl-1(tm5040)*, 11 ± 1 (n = 104); *sygl-1(q828)*, 11 ± 1 (n = 49)*; lst-1(ok814)*, 19 ± 2 (n = 20)*; lst-1(q826)*, 18 ± 2 (n = 23). (B) Total number of cells in PZ. The averages and standard deviations are as follows: wild type, 231 ± 33 (n = 12); *sygl-1(tm5040)*, 119 ± 17 (n = 22); *sygl-1(q828)*, 117 ± 16 (n = 20)*; lst-1(ok814)*, 207 ± 24 (n = 20)*; lst-1(q826)*, 192 ± 21 (n = 23). Box plot convention as in **Fig 2F**. Asterisks indicate a statistically significant difference by 1-way ANOVA with Tukey HSD *post hoc* test: ** p<0.001, * p<0.05, n.s. = non-significant. (C and D) Characterization of brood size, embryonic lethality, and fertility of *sygl-1* and *lst-1* mutants.
(TIF)

S3 Fig Characterization of *tbb-2* 3’UTR transgene. (A) Functionality of SYGL-1 protein encoded by the *tbb-2* 3’UTR transgene. (B-F) LST-1 expression in animals expressing varying abundance of SYGL-1. Assays are done with transgenic HA-tagged LST-1, which functions as endogenous LST-1 (**S1D Fig**). (B-E) Images of distal gonad stained with α-HA (LST-1, yellow) and DAPI (cyan), each a single z-slice. Conventions as in **Fig 1E–1J**; scale bar is 20 μm. Genotypes are: (B) *lst-1(ok814); qSi93[P*_*lst-1*_::*lst-1*::*1xHA*::*lst-1 3’end]*. (C) *lst-1(ok814) sygl-1(tm5040); qSi93[P*_*lst-1*_::*lst-1*::*1xHA*::*lst-1 3’end]*. (D) *lst-1(ok814) sygl-1(tm5040); qSi150[P*_*sygl-1*_::*3xFLAG*::*sygl-1*::*tbb-2 3’end]; qSi93[Pl*_*st-1*_::*lst-1*::*1xHA*::*lst-1 3’end]*. (E) wild type. (F) Total number of LST-1 expressing cells. Averages and standard deviations for each genotype are: (1) 48 ± 9 cells [5 ± 1 gcd] (n = 20); (2) 49 ± 9 cells [6 ± 1 gcd] (n = 20); (3) 48 ± 10 cells [5 ± 1 gcd] (n = 20). n.s. = non-significant by 1-way ANOVA with Tukey HSD *post hoc* test.
(TIF)

S4 Fig Characterization of *sygl-1(ubiq)* and *lst-1(ubiq)* tumors. (A and B) Penetrance of germline tumors in consecutive generations after removal from RNAi and at indicated temperatures, 15°C (pink), 20°C (green), 25°C (purple). Germline tumors scored by dissecting microscope after removal from *sygl-1* RNAi (A) or *lst-1* RNAi (B). Dots, mean values from at least 5 independent experiments; shaded areas, standard deviations. (C-H) Images of dissected young adult gonads stained with α-PGL-1 (white), α-FBF-1 (magenta), α-GLD-1 (green), and DAPI (cyan), each a single z-slice. (I-K) Images of dissected young male gonads stained with α-REC-8 (yellow), and DAPI (cyan). Conventions as in **Fig 1E–1J**; genotypes as detailed in **Fig 3E–3J**; scale bar is 20 μm.
(TIF)

S5 Fig Characterization of SYGL-1 and LST-1 in *fbf-1 fbf-2 sygl-1(ubiq)* and *fbf-1 fbf-2 lst-1(ubiq)* strains. (A-C) Dissected third larval stage (L3) gonads grown at 15°C before sperm differentiation, stained with α-FLAG (magenta) and DAPI (cyan). Shown are maximum z-projection images. Conventions and genotypes are as in **Fig 4G–4I**; scale bar is 20 μm. (D) Total germ cell number per gonadal arm, in each genotype. Total number of sperm in each gonad was converted to the number of germ cells for simplicity (see Methods). Loss of either *sygl-1* or *lst-1* enhances the GSC defect of *fbf-1 fbf-2*, as previously reported [25]. That loss is rescued by *sygl-1(ubiq)* or *lst-1(ubiq)*, confirming expression and functionality of SYGL-1 and LST-1 at 15°C. Box plot conventions as in **Fig 2F**. Averages and standard deviations for each genotype are as follows: (1) *fbf-1(ok91) fbf-2(q704)*, 26 ± 12, (n = 27); (2) *sygl-1(tm5040); fbf-1(ok91) fbf-2(q704)*, 17 ± 8 (n = 22); (3) *sygl-1(tm5040); fbf-1(ok91) fbf-2(q704) qSi235[Pmex-5*::*3xFLAG*::*sygl-1*::*tbb-2 3’end]*, 27 ± 8 (n = 17); (4) *lst-1(ok814); fbf-1(ok91) fbf-2(q704)*, 13 ± 8 (n = 18); (5) *lst-1(ok814); fbf-1(ok91) fbf-2(q704) qSi267[P*_*mex-5*_:: *lst-1*::*3xFLAG*::*tbb-2 3’end]*, 21 ± 10 (n = 20). Asterisks indicate a statistically significant difference by 1-way ANOVA with Tukey HSD *post hoc* test. ** p<0.001, * p<0.01, n.s. = non-significant. (E-I) Dissected young adult gonads raised at 25°C, stained with mitotic marker α-REC-8 (yellow), sperm marker α-SP56 (red), and DAPI (cyan). REC-8 localizes to the nucleus of mitotic germ cells but is diffuse in meiotic germ cells [30]. Conventions and genotypes are as in **Fig 4G–4I**; images are a single z-slice, scale bar is 20 μm. Germlines in *fbf-1 fbf-2* mutant adults can proliferate at 25°C, as previously reported [40]. Loss of either *sygl-1* or *lst-1* enhances the GSC defects of *fbf-1 fbf-2* [25; this work]. That loss is rescued by *sygl-1(ubiq)* or *lst-1(ubiq)*, confirming expression and functionality of SYGL-1(ubiq) and LST-1(ubiq) at 25°C. Regardless, SYGL-1(ubiq) and LST-1(ubiq) do not generate germline tumors. (J) Summary of epistasis test with *fbf-1 fbf-2* at 25°C. Asterisks indicate a statistically significant difference by 1-way ANOVA with Tukey HSD *post hoc* test. ** p<0.01, n.s. = non-significant.
(TIF)

S6 Fig *sygl-1* and *lst-1* are not required for FBF expression. (A-F) Dissected young adult gonads stained with α-FLAG (FBF-1 or FBF-2, magenta) and DAPI (cyan). FBF-1 (A-C) or FBF-2 (D-F) was measured with and without *sygl-1* and *lst-1*. All experiments were done in *gld-2 gld-1* tumorous germlines to compare cells in the same state. Genotypes: (A) *gld-2(q497) gld-1(q485); fbf-1(ok91) qSi232[P*_*fbf-1*_::*3xFLAG*::*fbf-1*::*fbf-1 3’end]*. (B) *lst-1(ok814) sygl-1(tm5040) gld-2(q497) gld-1(q485); fbf-1(ok91) qSi232[P*_*fbf-1*_::*3xFLAG*::*fbf-1*::*fbf-1 3’end]*. (C) wild type. (D) *gld-2(q497) gld-1(q485); fbf-2(q738) qSi75[P*_*fbf-2*_::*3xFLAG*::*fbf-2*::*fbf-2 3’end]*. (E) *lst-1(ok814) sygl-1(tm5040) gld-2(q497) gld-1(q485); fbf-2(q738) qSi75[P*_*fbf-2*_::*3xFLAG*::*fbf-2*::*fbf-2 3’end]*. (F) wild type. All images are maximum intensity z-projections. Conventions as in **Fig 1E–1J**; scale bar is 20 μm. (G) Western blots. Blot was probed with α-FLAG (FBF-1 or FBF-2) or α-actin, and the ratio between α-FLAG and α-actin was calculated. FBF-1 was expressed at similar abundance with and without SYGL-1 and LST-1, whereas a minor increase of FBF-2 was observed without SYGL-1 and LST-1. This minor effect may reflect indirect regulation between *sygl-1*, *lst-1* and *fbf-2*, perhaps a by-product of their role in the genetic circuity. Genotypes: (1) *gld-2(q497) gld-1(q485); fbf-1(ok91) qSi232[P*_*fbf-1*_::*3xFLAG*::*fbf-1*::*fbf-1 3’end]*. (2) *lst-1(ok814) sygl-1(tm5040) gld-2(q497) gld-1(q485); fbf-1(ok91) qSi232[P*_*fbf-1*_::*3xFLAG*::*fbf-1*::*fbf-1 3’end]*. (3) *gld-2(q497) gld-1(q485); fbf-2(q738) qSi75[P*_*fbf-2*_::*3xFLAG*::*fbf-2*::*fbf-2 3’end]*. (4) *lst-1(ok814) sygl-1(tm5040) gld-2(q497) gld-1(q485); fbf-2(q738) qSi75[P*_*fbf-2*_::*3xFLAG*::*fbf-2*::*fbf-2 3’end]*. (5) wild type. (6) *fbf-1(ok91) qSi232[P*_*fbf-1*_::*3xFLAG*::*fbf-1*::*fbf-1 3’end]*. (7) *fbf-2(q738) qSi75[P*_*fbf-2*_::*3xFLAG*::*fbf-2*::*fbf-2 3’end]*.
(TIF)

S7 Fig 3xV5::FBF-2 is a functional protein. (A) Schematic of *fbf-2* endogenous locus. Conventions as in **Fig 1C**. 3xV5 epitope tag was inserted at the N-terminus of *fbf-2* to generate *fbf-2(q932)*. The *fbf-2(q738)* deletion is a loss-of-function allele [81]. (B) Progenitor zone (PZ) lengths were measured in germ cell diameters from the distal end (gcd). The *fbf-2(q738)* deletion mutant has an increased PZ size, as previously reported [81]. The PZ length of *fbf-2(q932)* is indistinguishable from wild type; 3xV5::FBF-2 is therefore functional. Box plot conventions as in **Fig 2F**. Averages and standard deviations for each genotype are as follows: (1) wild type, 19 ± 2 (n = 13); (2) *fbf-2(q932)*, 19 ± 2 (n = 25); (3) *fbf-2(q738)*, 27 ± 2 (n = 35). Asterisks indicate a statistically significant difference by 1-way ANOVA with Tukey HSD *post hoc* test. ** p<0.001, n.s. = non-significant. (C and D) Images of distal gonads stained with α-V5 (FBF-2, magenta) and DAPI (cyan), each a single z-slice. Genotypes: *fbf-2(q932)* (C), wild type (D). Conventions as in **Fig 1E–1J**; scale bar is 20 μm.
(TIF)

S8 Fig *gld-1* smFISH probe set is specific to *gld-1* mRNA. (A) The *gld-1(q485)* deletion causes a frameshift and thus a null phenotype [45]. (B-E) Dissected gonads probed for *gld-1* smFISH probe (white) and DAPI (cyan). (B and C) wild type; (D and E) *gld-2(q497) gld-1(q485)*. (C and E) Boxed areas in B and D were magnified in C and E respectively to reveal *gld-1* nascent transcripts in the nucleus (pink arrows) and *gld-1* mature mRNAs in the cytoplasm (yellow arrowheads). Top, *gld-1* RNAs; Bottom, RNAs merged with DAPI. Images are maximum intensity z-projection (B and D), or a single slice (C and E). Conventions as in **Fig 1E–1J**; scale bar is 20μm (B and D) or 2 μm (C and E).
(TIF)

S1 Table Nematode strains used in this study. (PDF)

S2 Table MosSCI transgenes generated in this study. (PDF)

S3 Table CRISPR alleles generated in this study. (PDF)

S4 Table Plasmids used to generate CRISPR and MosSCI transgenes. (PDF)

S5 Table Sequences of crRNA and repair oligos used to generate CRISPR alleles. (PDF)
