# Supplementary figures and images for: SYGL-1 and LST-1 link niche signaling to PUF RNA repression for stem cell maintenance in Caenorhabditis elegans
Source: PLoS Genet. 2017 Dec 12;13(12):e1007121. doi: 10.1371/journal.pgen.1007121 (PMC5741267; doi:10.1371/journal.pgen.1007121)

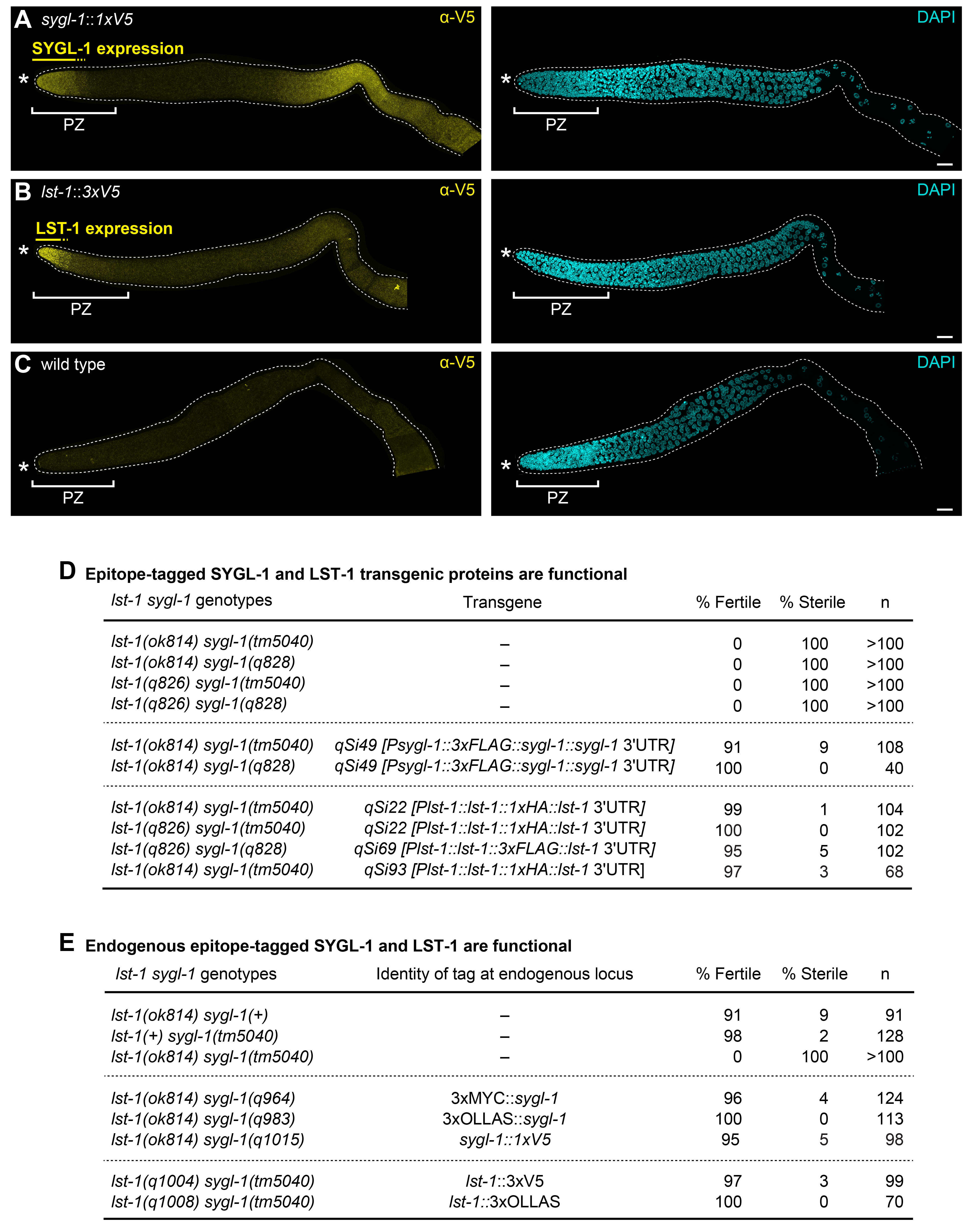

Supplement: S1 Fig — (A-C) SYGL-1 and LST-1 in dissected gonads. Representative z-projection images of staining with α-V5, using sygl-1::1xV5 and lst-1::3xV5 epitope tagged alleles. Conventions are as in Fig 1E–1J; scale bar is 20 μm. Genotypes are (A) sygl-1(q1015)[sygl-1::1xV5], (B) lst-1(q1004)[lst-1::3xV5], (C) wild type. In addition to distal expression within the progenitor zone (PZ), SYGL-1 and LST-1 are present in the proximal gonad, consistent with their mRNA expression [18, 19]. (D and E) Functionality of epitope-tagged SYGL-1 or LST-1 transgenic proteins (D) or endogenous alleles (E). Because lst-1 sygl-1 double mutants are 100% sterile but single mutants are fertile [18], functionality of epitope-tagged transgenes or endogenous alleles was tested by scoring fertility in the appropriate mutant background. (TIF) [file pgen.1007121.s001.tif]

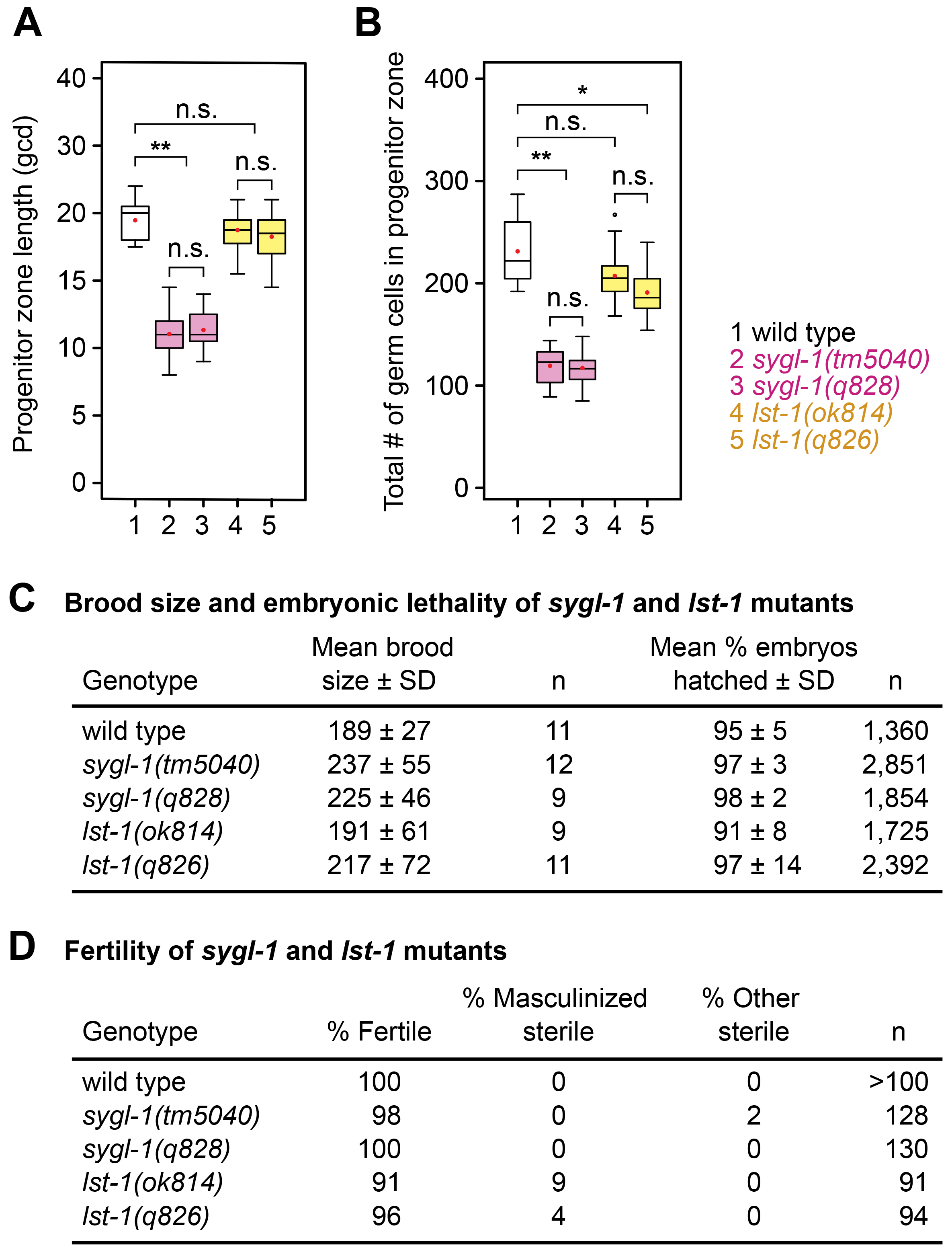

Supplement: S2 Fig — (A and B) Progenitor zone (PZ) size in sygl-1 and lst-1 mutants. (A) PZ length measured in number of germ cell diameters (gcd) from distal end. The averages and standard deviations are as follows: wild type, 19 ± 2 (n = 13); sygl-1(tm5040), 11 ± 1 (n = 104); sygl-1(q828), 11 ± 1 (n = 49); lst-1(ok814), 19 ± 2 (n = 20); lst-1(q826), 18 ± 2 (n = 23). (B) Total number of cells in PZ. The averages and standard deviations are as follows: wild type, 231 ± 33 (n = 12); sygl-1(tm5040), 119 ± 17 (n = 22); sygl-1(q828), 117 ± 16 (n = 20); lst-1(ok814), 207 ± 24 (n = 20); lst-1(q826), 192 ± 21 (n = 23). Box plot convention as in Fig 2F. Asterisks indicate a statistically significant difference by 1-way ANOVA with Tukey HSD post hoc test: ** p<0.001, * p<0.05, n.s. = non-significant. (C and D) Characterization of brood size, embryonic lethality, and fertility of sygl-1 and lst-1 mutants. (TIF) [file pgen.1007121.s002.tif]

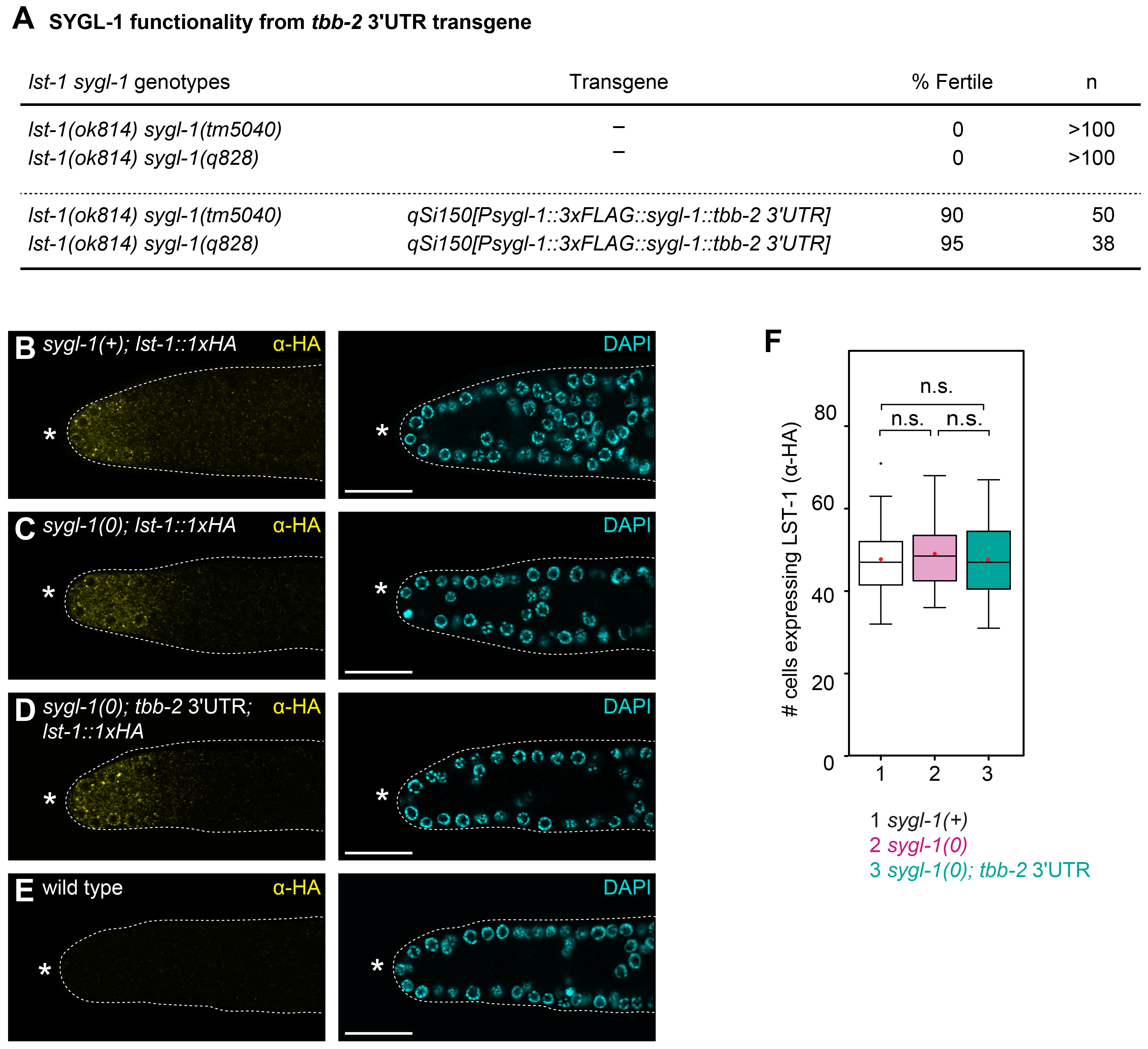

Supplement: S3 Fig — (A) Functionality of SYGL-1 protein encoded by the tbb-2 3’UTR transgene. (B-F) LST-1 expression in animals expressing varying abundance of SYGL-1. Assays are done with transgenic HA-tagged LST-1, which functions as endogenous LST-1 (S1D Fig). (B-E) Images of distal gonad stained with α-HA (LST-1, yellow) and DAPI (cyan), each a single z-slice. Conventions as in Fig 1E–1J; scale bar is 20 μm. Genotypes are: (B) lst-1(ok814); qSi93[Plst-1::lst-1::1xHA::lst-1 3’end]. (C) lst-1(ok814) sygl-1(tm5040); qSi93[Plst-1::lst-1::1xHA::lst-1 3’end]. (D) lst-1(ok814) sygl-1(tm5040); qSi150[Psygl-1::3xFLAG::sygl-1::tbb-2 3’end]; qSi93[Plst-1::lst-1::1xHA::lst-1 3’end]. (E) wild type. (F) Total number of LST-1 expressing cells. Averages and standard deviations for each genotype are: (1) 48 ± 9 cells [5 ± 1 gcd] (n = 20); (2) 49 ± 9 cells [6 ± 1 gcd] (n = 20); (3) 48 ± 10 cells [5 ± 1 gcd] (n = 20). n.s. = non-significant by 1-way ANOVA with Tukey HSD post hoc test. (TIF) [file pgen.1007121.s003.tif]

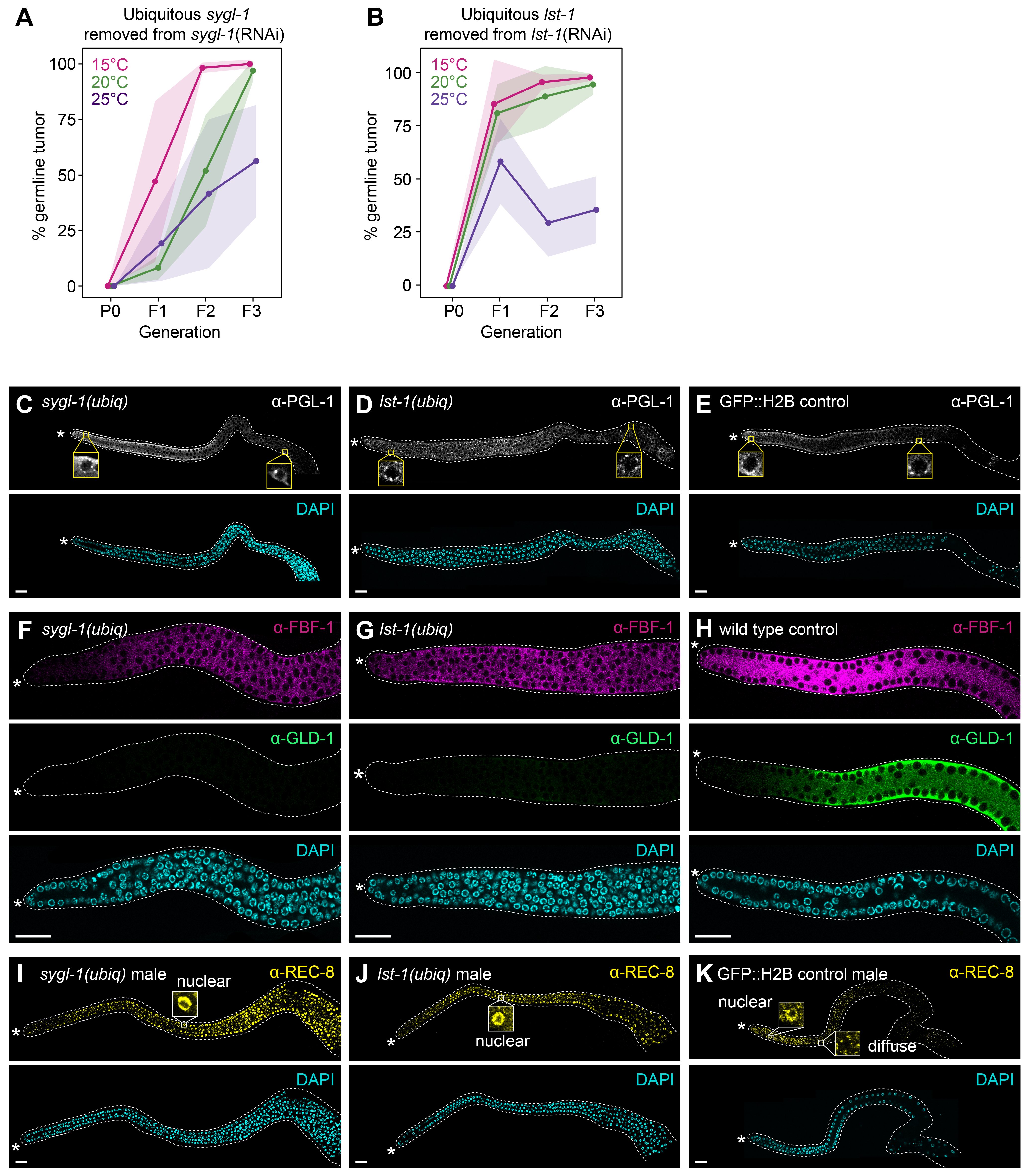

Supplement: S4 Fig — (A and B) Penetrance of germline tumors in consecutive generations after removal from RNAi and at indicated temperatures, 15°C (pink), 20°C (green), 25°C (purple). Germline tumors scored by dissecting microscope after removal from sygl-1 RNAi (A) or lst-1 RNAi (B). Dots, mean values from at least 5 independent experiments; shaded areas, standard deviations. (C-H) Images of dissected young adult gonads stained with α-PGL-1 (white), α-FBF-1 (magenta), α-GLD-1 (green), and DAPI (cyan), each a single z-slice. (I-K) Images of dissected young male gonads stained with α-REC-8 (yellow), and DAPI (cyan). Conventions as in Fig 1E–1J; genotypes as detailed in Fig 3E–3J; scale bar is 20 μm. (TIF) [file pgen.1007121.s004.tif]

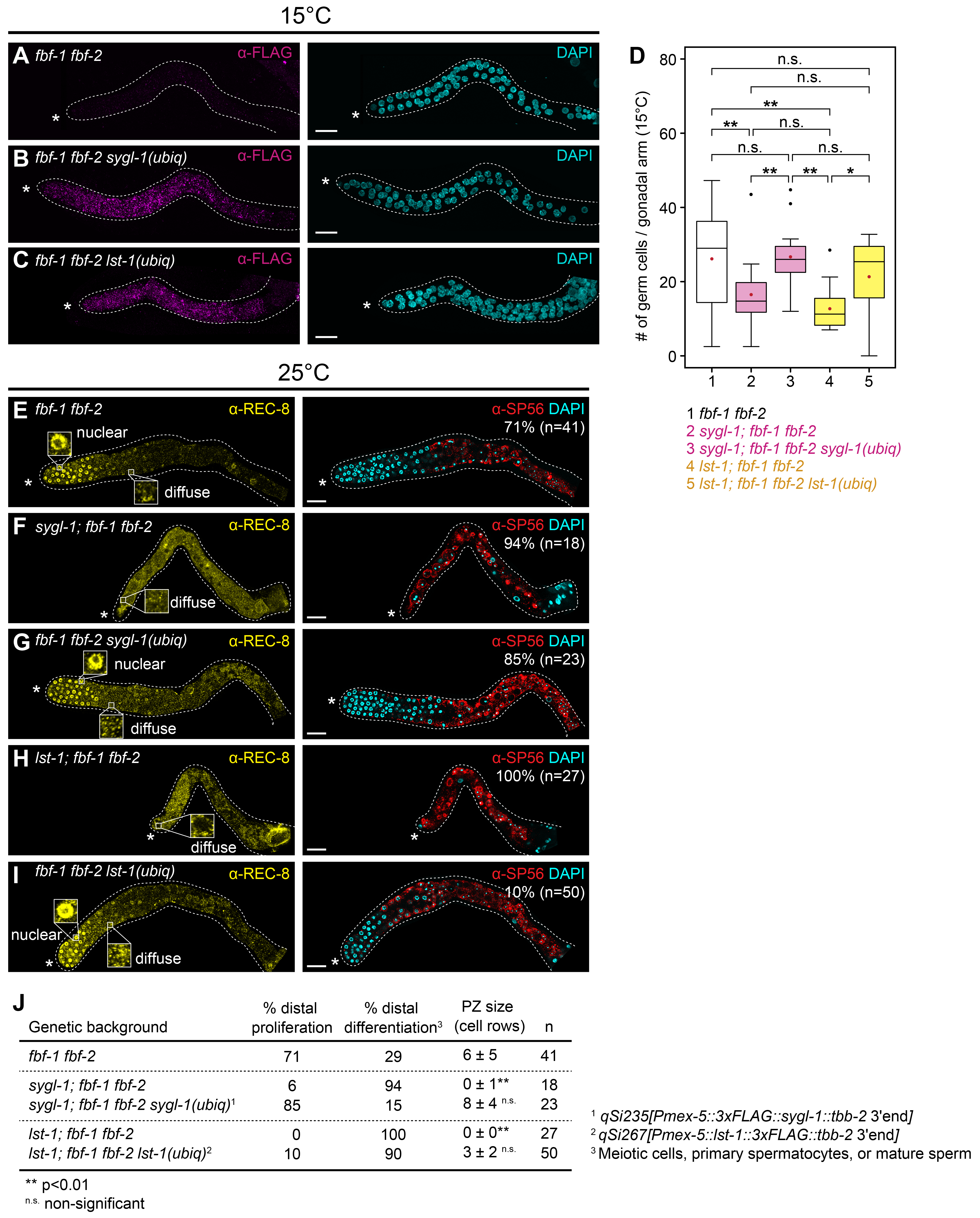

Supplement: S5 Fig — (A-C) Dissected third larval stage (L3) gonads grown at 15°C before sperm differentiation, stained with α-FLAG (magenta) and DAPI (cyan). Shown are maximum z-projection images. Conventions and genotypes are as in Fig 4G–4I; scale bar is 20 μm. (D) Total germ cell number per gonadal arm, in each genotype. Total number of sperm in each gonad was converted to the number of germ cells for simplicity (see Methods). Loss of either sygl-1 or lst-1 enhances the GSC defect of fbf-1 fbf-2, as previously reported [25]. That loss is rescued by sygl-1(ubiq) or lst-1(ubiq), confirming expression and functionality of SYGL-1 and LST-1 at 15°C. Box plot conventions as in Fig 2F. Averages and standard deviations for each genotype are as follows: (1) fbf-1(ok91) fbf-2(q704), 26 ± 12, (n = 27); (2) sygl-1(tm5040); fbf-1(ok91) fbf-2(q704), 17 ± 8 (n = 22); (3) sygl-1(tm5040); fbf-1(ok91) fbf-2(q704) qSi235[Pmex-5::3xFLAG::sygl-1::tbb-2 3’end], 27 ± 8 (n = 17); (4) lst-1(ok814); fbf-1(ok91) fbf-2(q704), 13 ± 8 (n = 18); (5) lst-1(ok814); fbf-1(ok91) fbf-2(q704) qSi267[Pmex-5:: lst-1::3xFLAG::tbb-2 3’end], 21 ± 10 (n = 20). Asterisks indicate a statistically significant difference by 1-way ANOVA with Tukey HSD post hoc test. ** p<0.001, * p<0.01, n.s. = non-significant. (E-I) Dissected young adult gonads raised at 25°C, stained with mitotic marker α-REC-8 (yellow), sperm marker α-SP56 (red), and DAPI (cyan). REC-8 localizes to the nucleus of mitotic germ cells but is diffuse in meiotic germ cells [30]. Conventions and genotypes are as in Fig 4G–4I; images are a single z-slice, scale bar is 20 μm. Germlines in fbf-1 fbf-2 mutant adults can proliferate at 25°C, as previously reported [40]. Loss of either sygl-1 or lst-1 enhances the GSC defects of fbf-1 fbf-2 [25; this work]. That loss is rescued by sygl-1(ubiq) or lst-1(ubiq), confirming expression and functionality of SYGL-1(ubiq) and LST-1(ubiq) at 25°C. Regardless, SYGL-1(ubiq) and LST-1(ubiq) do not generate germline tumors. (J) Summar [file pgen.1007121.s005.tif]

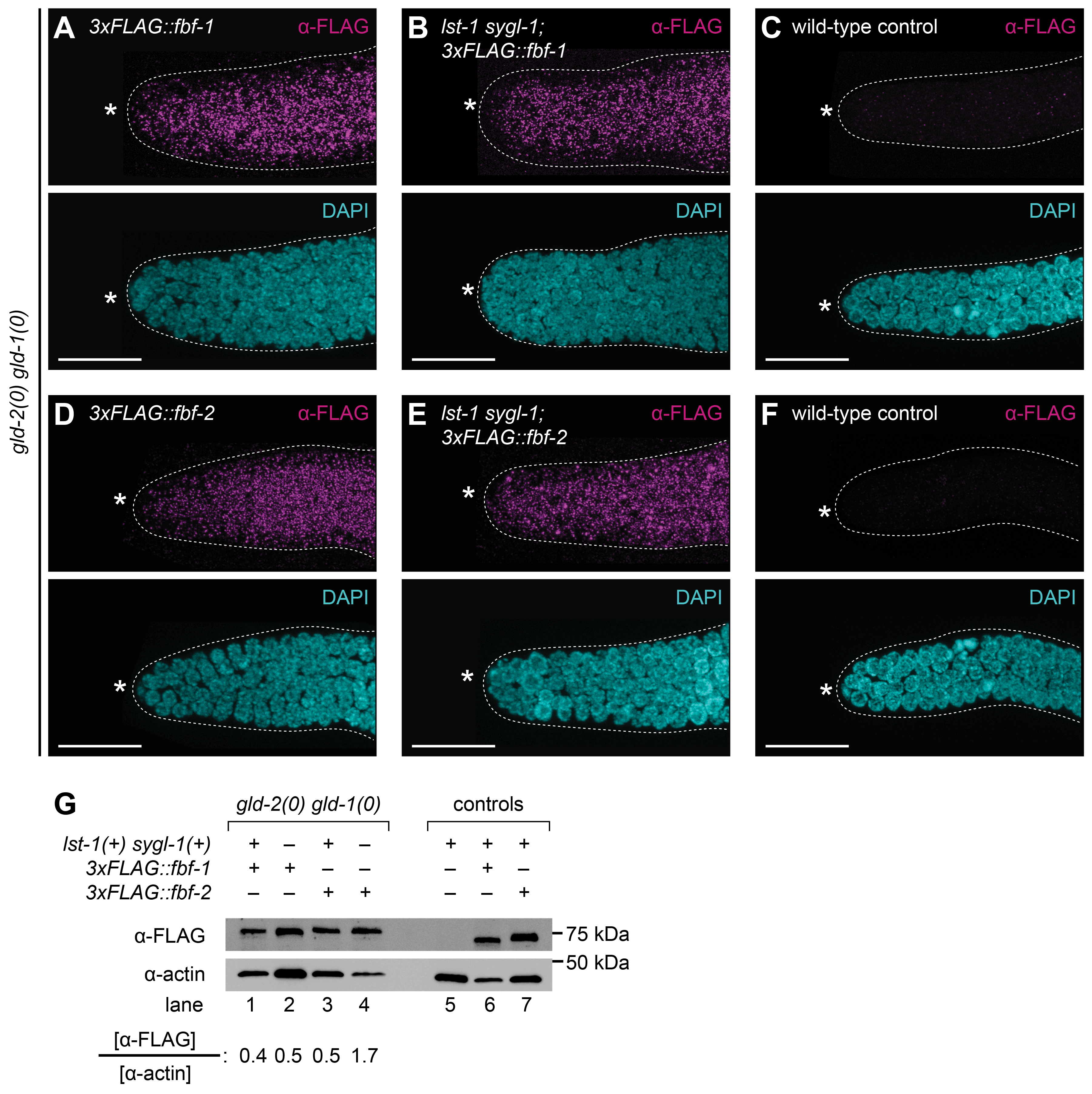

Supplement: S6 Fig — (A-F) Dissected young adult gonads stained with α-FLAG (FBF-1 or FBF-2, magenta) and DAPI (cyan). FBF-1 (A-C) or FBF-2 (D-F) was measured with and without sygl-1 and lst-1. All experiments were done in gld-2 gld-1 tumorous germlines to compare cells in the same state. Genotypes: (A) gld-2(q497) gld-1(q485); fbf-1(ok91) qSi232[Pfbf-1::3xFLAG::fbf-1::fbf-1 3’end]. (B) lst-1(ok814) sygl-1(tm5040) gld-2(q497) gld-1(q485); fbf-1(ok91) qSi232[Pfbf-1::3xFLAG::fbf-1::fbf-1 3’end]. (C) wild type. (D) gld-2(q497) gld-1(q485); fbf-2(q738) qSi75[Pfbf-2::3xFLAG::fbf-2::fbf-2 3’end]. (E) lst-1(ok814) sygl-1(tm5040) gld-2(q497) gld-1(q485); fbf-2(q738) qSi75[Pfbf-2::3xFLAG::fbf-2::fbf-2 3’end]. (F) wild type. All images are maximum intensity z-projections. Conventions as in Fig 1E–1J; scale bar is 20 μm. (G) Western blots. Blot was probed with α-FLAG (FBF-1 or FBF-2) or α-actin, and the ratio between α-FLAG and α-actin was calculated. FBF-1 was expressed at similar abundance with and without SYGL-1 and LST-1, whereas a minor increase of FBF-2 was observed without SYGL-1 and LST-1. This minor effect may reflect indirect regulation between sygl-1, lst-1 and fbf-2, perhaps a by-product of their role in the genetic circuity. Genotypes: (1) gld-2(q497) gld-1(q485); fbf-1(ok91) qSi232[Pfbf-1::3xFLAG::fbf-1::fbf-1 3’end]. (2) lst-1(ok814) sygl-1(tm5040) gld-2(q497) gld-1(q485); fbf-1(ok91) qSi232[Pfbf-1::3xFLAG::fbf-1::fbf-1 3’end]. (3) gld-2(q497) gld-1(q485); fbf-2(q738) qSi75[Pfbf-2::3xFLAG::fbf-2::fbf-2 3’end]. (4) lst-1(ok814) sygl-1(tm5040) gld-2(q497) gld-1(q485); fbf-2(q738) qSi75[Pfbf-2::3xFLAG::fbf-2::fbf-2 3’end]. (5) wild type. (6) fbf-1(ok91) qSi232[Pfbf-1::3xFLAG::fbf-1::fbf-1 3’end]. (7) fbf-2(q738) qSi75[Pfbf-2::3xFLAG::fbf-2::fbf-2 3’end]. (TIF) [file pgen.1007121.s006.tif]

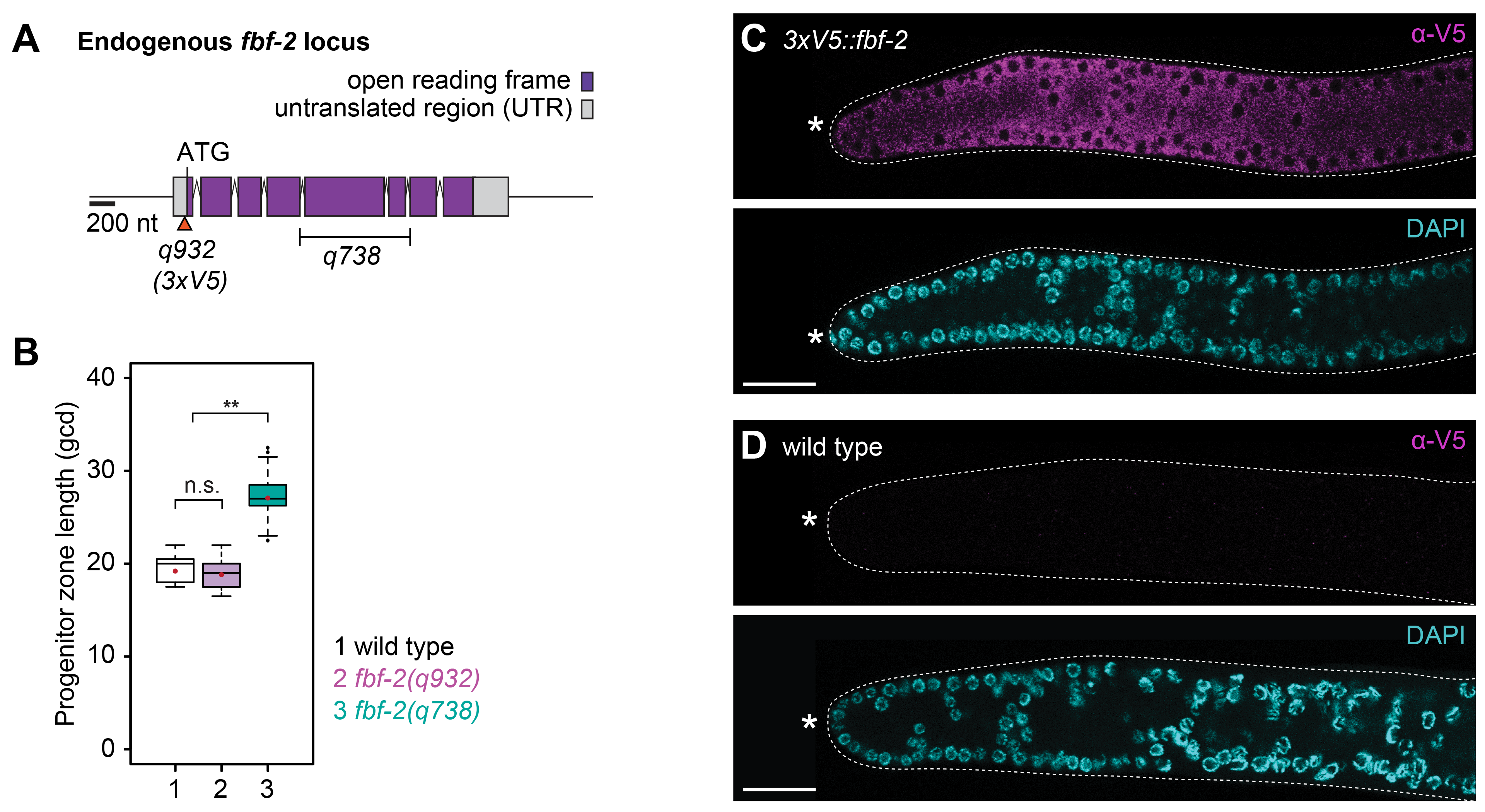

Supplement: S7 Fig — (A) Schematic of fbf-2 endogenous locus. Conventions as in Fig 1C. 3xV5 epitope tag was inserted at the N-terminus of fbf-2 to generate fbf-2(q932). The fbf-2(q738) deletion is a loss-of-function allele [81]. (B) Progenitor zone (PZ) lengths were measured in germ cell diameters from the distal end (gcd). The fbf-2(q738) deletion mutant has an increased PZ size, as previously reported [81]. The PZ length of fbf-2(q932) is indistinguishable from wild type; 3xV5::FBF-2 is therefore functional. Box plot conventions as in Fig 2F. Averages and standard deviations for each genotype are as follows: (1) wild type, 19 ± 2 (n = 13); (2) fbf-2(q932), 19 ± 2 (n = 25); (3) fbf-2(q738), 27 ± 2 (n = 35). Asterisks indicate a statistically significant difference by 1-way ANOVA with Tukey HSD post hoc test. ** p<0.001, n.s. = non-significant. (C and D) Images of distal gonads stained with α-V5 (FBF-2, magenta) and DAPI (cyan), each a single z-slice. Genotypes: fbf-2(q932) (C), wild type (D). Conventions as in Fig 1E–1J; scale bar is 20 μm. (TIF) [file pgen.1007121.s007.tif]

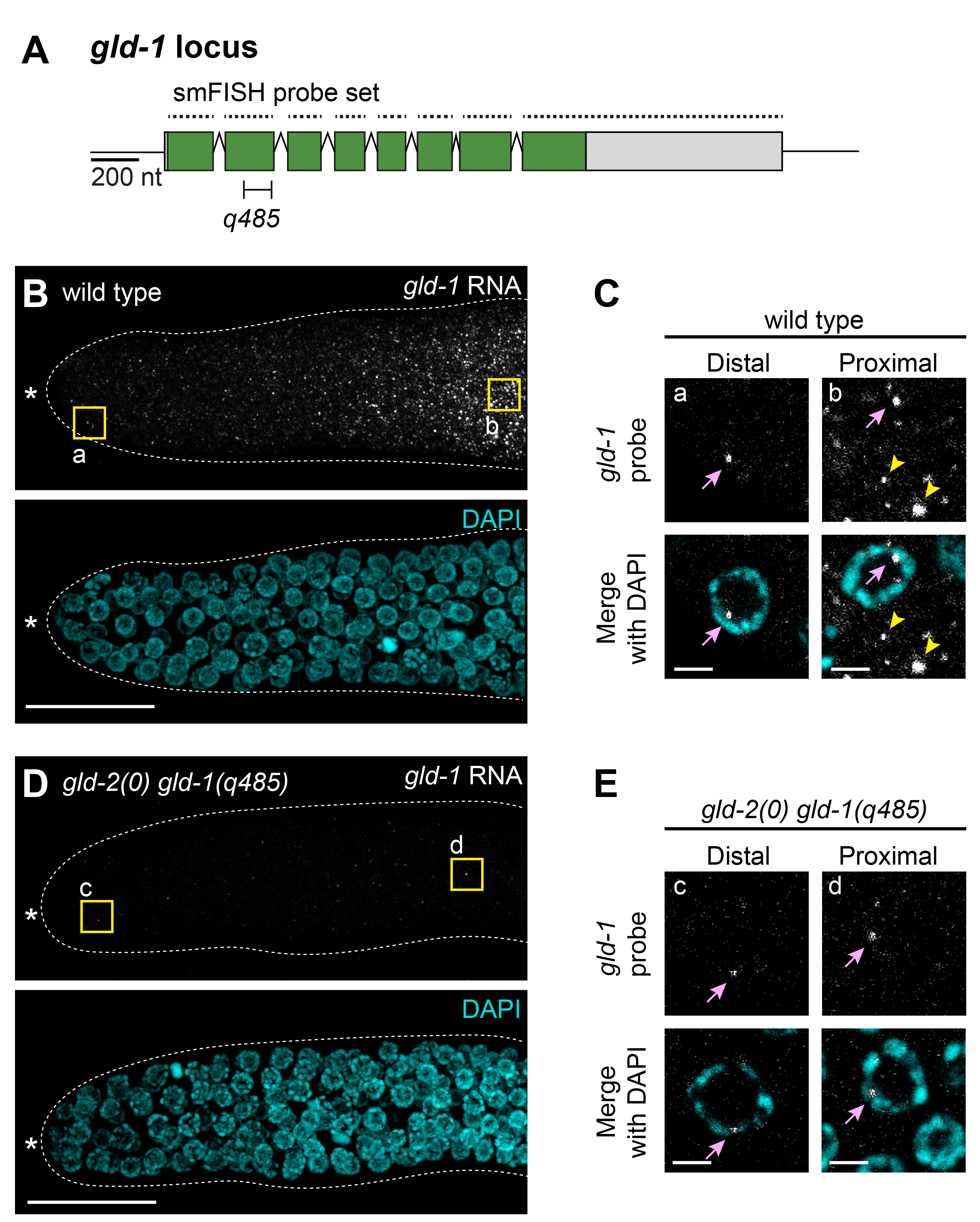

Supplement: S8 Fig — (A) The gld-1(q485) deletion causes a frameshift and thus a null phenotype [45]. (B-E) Dissected gonads probed for gld-1 smFISH probe (white) and DAPI (cyan). (B and C) wild type; (D and E) gld-2(q497) gld-1(q485). (C and E) Boxed areas in B and D were magnified in C and E respectively to reveal gld-1 nascent transcripts in the nucleus (pink arrows) and gld-1 mature mRNAs in the cytoplasm (yellow arrowheads). Top, gld-1 RNAs; Bottom, RNAs merged with DAPI. Images are maximum intensity z-projection (B and D), or a single slice (C and E). Conventions as in Fig 1E–1J; scale bar is 20μm (B and D) or 2 μm (C and E). (TIF) [file pgen.1007121.s008.tif]
