# Supplementary material for: SYGL-1 and LST-1 link niche signaling to PUF RNA repression for stem cell maintenance in Caenorhabditis elegans
Source: PLoS Genet. 2017 Dec 12;13(12):e1007121. doi: 10.1371/journal.pgen.1007121 (PMC5741267; doi:10.1371/journal.pgen.1007121)
Supplement: S1 Table — (PDF) [file pgen.1007121.s009.pdf]

S1 Table. Nematode strains used in this study

Name	Genotype	Reference
N2	wild type	*Brenner, 1974*
DG627	*emb-30(tn377ts) III*	*Furata et al, 2000*
EG4322	*ttTi5605 II; unc-119(ed3) III*	*Frøkjær-Jensen et al, 2008*
EG6699	*ttTi5605 II; unc-119(ed3) III*	*Frøkjær-Jensen et al, 2012*
EG6703	*unc-119(ed3) III; cxTi10816 IV; oxEx1582*	*Frøkjær-Jensen et al, 2012*
HT1593	*unc-119(ed3) III*	*Maduro and Pilgrim, 1995*
JA1515	*weSi2[P_{mex-5}::GFP::his-58::tbb-2 3'end] II; unc-119(ed3) III*	*Zeiser et al, 2011*
JK4299	*gld-2(q497) gld-1(q361) I/ hT2[qIs48](I;III)*	*This work*
JK4356	*lst-1(ok814) I*	*Singh et al, 2011*
JK4361	*lst-1(ok814) I; fbf-1(ok91) fbf-2(q704)/ mIn1[mIs14 dpy-10(e128)] II*	*This work*
JK4774	*lst-1(ok814) sygl-1(tm5040) I/ hT2[qIs48](I;III)*	*Kershner et al, 2014*
JK4836	*lst-1(ok814) I; qSi22[P_{lst-1}::lst-1::1xHA::lst-1 3'end] II*	*This work*
JK4852	*lst-1(ok814) sygl-1(tm5040) I; qSi22[P_{lst-1}::lst-1::1xHA::lst-1 3'end] II*	*This work*
JK4862	*glp-1(q46) III/ hT2[qIs48](I;III)*	*Kershner et al, 2014*
JK4942	*sygl-1(tm5040) I; qSi49[P_{sygl-1}::3xFLAG::sygl-1::sygl-1 3'end] II; unc-119(ed3) III*	*This work*
JK4950	*lst-1(ok814) I; ttTi5605 II; unc-119(ed3) III*	*This work*
JK4966	*sygl-1(tm5040) I; ttTi5605 II; unc-119(ed3) III*	*This work*
JK4996	*lst-1(ok814) I; qSi69[P_{lst-1}::lst-1::3xFLAG::lst-1 3'end] II; unc-119(ed3) III*	*This work*
JK5064	*sygl-1(tm5040) I; fbf-1(ok91) fbf-2(q704)/ mIn1[mIs14 dpy-10(e128)] II*	*This work*

JK5073	*lst-1(ok814) sygl-1(tm5040) I; qSi93[*P_{lst-1}*::lst-1::1xHA::lst-1 3'end] IV*	*This work*
JK5195	*sygl-1(tm5040) I; qSi150[*P_{sygl-1}*::3xFLAG::sygl-1::tbb-2 3'end] II; unc-119(ed3) III*	*This work*
JK5205	*lst-1(ok814) I; qSi93[*P_{lst-1}*::lst-1::1xHA::lst-1 3'end] IV*	*This work*
JK5233	*sygl-1(tm5040) I/ hT2[qIs48](I;III); qSi150[*P_{sygl-1}*::3xFLAG::sygl-1::tbb-2 3'end] II; emb-30(tn377ts) III/ hT2[qIs48](I;III)*	*This work*
JK5235	*sygl-1(tm5040) I/ hT2[qIs48](I;III); emb-30(tn377ts) III/ hT2[qIs48](I;III)*	*This work*
JK5263	*lst-1(ok814) sygl-1(tm5040) I/ hT2[qIs48](I;III); qSi150[*P_{sygl-1}*::3xFLAG::sygl-1::tbb-2 3'end] II; qSi93[*P_{lst-1}*::lst-1::1xHA::lst-1 3'end] IV*	*This work*
JK5277	*lst-1(q826) I/ hT2[qIs48](I;III)*	*This work*
JK5315	*lst-1(q826) sygl-1(tm5040) I/ hT2[qIs48](I;III)*	*This work*
JK5366	*sygl-1(tm5040) I; qSi235[*P_{mex-5}*::3xFLAG::sygl-1::tbb-2 3'end] II; unc-119(ed3) III*	*This work*
JK5401	*sygl-1(tm5040) I/ hT2[qIs48](I;III); qSi235[*P_{mex-5}*::3xFLAG::sygl-1::tbb-2 3'end] II; glp-1(q46) III/ hT2[qIs48](I;III)*	*This work*
JK5403	*lst-1(ok814) sygl-1(tm5040) I/ hT2[qIs48](I;III); qSi235[*P_{mex-5}*::3xFLAG::sygl-1::tbb-2 3'end] II*	*This work*
JK5411	*sygl-1(tm5040) I; fbf-1(ok91) fbf-2(q704) qSi235[*P_{mex-5}*::3xFLAG::sygl-1::tbb-2 3'end]/ mIn1[mIs14 dpy-10(e128)] II*	*This work*
JK5485	*lst-1(ok814) I; qSi267[*P_{mex-5}*::lst-1::3xFLAG::tbb-2 3'end] II; unc-119(ed3) III*	*This work*
JK5499	*sygl-1(q828) I; qSi49[*P_{sygl-1}*::3xFLAG::sygl-1::sygl-1 3'end] II*	*This work*
JK5500	*sygl-1(q828) I; qSi150[*P_{sygl-1}*::3xFLAG::sygl-1::tbb-2 3'end] II*	*This work*
JK5537	*lst-1(ok814) I; fbf-1(ok91) fbf-2(q704) qSi267[*P_{mex-5}*::lst-1::3xFLAG::tbb-2 3'end]/ mIn1[mIs14 dpy-10(e128)] II*	*This work*
JK5538	*lst-1(ok814) I/ hT2[qIs48](I;III); qSi267[*P_{mex-5}*::lst-1::3xFLAG::tbb-2 3'end] II; glp-1(q46) III/ hT2[qIs48](I;III)*	*This work*
JK5574	*sygl-1(tm5040) I; qSi297[*P_{mex-5}*::3xMYC::sygl-1::tbb-2 3'end] II; unc-119(ed3) III*	*This work*
JK5585	*lst-1(ok814) sygl-1(tm5040) I/ hT2[qIs48](I;III); qSi267[*P_{mex-5}*::lst-1::3xFLAG::tbb-2 3'end] II*	*This work*
JK5590	*lst-1(ok814) sygl-1(q828) I/ hT2[qIs48](I;III)*	*This work*
JK5600	*gld-2(q497) gld-1(q485) I/ hT2[qIs48](I;III); fbf-1(ok91) qSi232[*P_{fbf-1}*::3xFLAG::fbf-1::fbf-1 3'end] II*	*This work*
JK5602	*gld-2(q497) gld-1(q485) I/ hT2[qIs48](I;III); fbf-2(q738) qSi75[*P_{fbf-2}*::3xFLAG::fbf-2::fbf-2 3'end] II*	*This work*

JK5603	*gld-2(q497) gld-1(q485) lst-1(ok814) sygl-1(tm5040) I/ hT2[qIs48](I;III); fbf-1(ok91) qSi232[P_{fbf-1}::3xFLAG::fbf-1::fbf-1 3'end] II*	*This work*
JK5604	*gld-2(q497) gld-1(q485) lst-1(ok814) sygl-1(tm5040) I/ hT2[qIs48](I;III); fbf-2(q738) qSi75[P_{fbf-2}::3xFLAG::fbf-2::fbf-2 3'end] II*	*This work*
JK5621	*sygl-1(tm5040) I*	*This work*
JK5622	*sygl-1(q828) I*	*This work*
JK5623	*lst-1(ok814) sygl-1(tm5040) I; qSi49[P_{sygl-1}::3xFLAG::sygl-1::sygl-1 3'end] II*	*This work*
JK5624	*lst-1(ok814) sygl-1(q828) I; qSi49[P_{sygl-1}::3xFLAG::sygl-1::sygl-1 3'end] II*	*This work*
JK5625	*lst-1(ok814) sygl-1(q828) I; qSi150[P_{sygl-1}::3xFLAG::sygl-1::tbb-2 3'end] II*	*This work*
JK5656	*lst-1(ok814) sygl-1(tm5040) I; qSi150[P_{sygl-1}::3xFLAG::sygl-1::tbb-2 3'end] II*	*This work*
JK5760	*lst-1(ok814) sygl-1(q828) gld-2(q497) gld-1(q361) I/ hT2[qIs48](I;III)*	*This work*
JK5761	*sygl-1(q828) gld-2(q497) gld-1(q361) I/ hT2[qIs48](I;III)*	*This work*
JK5762	*lst-1(ok814) gld-2(q497) gld-1(q361) I/ hT2[qIs48](I;III)*	*This work*
JK5783	*sygl-1(tm5040) I; fbf-2(q931)[3xV5::fbf-2] qSi235[P_{mex-5}::3xFLAG::sygl-1::tbb-2 3'end] II*	*This work*
JK5842	*fbf-2(q932)[3xV5::fbf-2] II*	*This work*
JK5844	*sygl-1(tm5040) I; fbf-2(q932)[3xV5::fbf-2] qSi297[P_{mex-5}::3xMYC::sygl-1::tbb-2 3'end] II*	*This work*
JK5850	*sygl-1(q964)[3xMYC::sygl-1] I*	*This work*
JK5867	*lst-1(q826) sygl-1(q828) I/ hT2[qIs48](I;III)*	*This work*
JK5893	*sygl-1(q983)[3xOLLAS::sygl-1] I*	*This work*
JK5929	*lst-1(q1004)[lst-1::3xV5] I*	*This work*
JK5930	*lst-1(ok814) sygl-1(q964)[3xMYC::sygl-1] I*	*This work*
JK5934	*lst-1(q826) sygl-1(q828) I; qSi69[P_{lst-1}::lst-1::3xFLAG::lst-1 3'end] II*	*This work*
JK5937	*fog-3(q520) I/ hT2[qIs48](I;III); qSi235[P_{mex-5}::3xFLAG::sygl-1::tbb-2 3'end] II*	*This work*
JK5938	*fog-3(q520) I/ hT2[qIs48](I;III); qSi267[P_{mex-5}::lst-1::3xFLAG::tbb-2 3'end] II*	*This work*

JK5948	*lst-1(q1004)[lst-1::3xV5] sygl-1(tm5040) I*	*This work*
JK5968	*lst-1(ok814) sygl-1(q983)[3xOLLAS::sygl-1] I*	*This work*
JK5964	*lst-1(q1008)[lst-1::3xOLLAS] I*	*This work*
JK6002	*sygl-1(q1015)[sygl-1::1xV5] I*	*This work*
JK6008	*lst-1(ok814) sygl-1(q1015)[sygl-1::1xV5] I*	*This work*
JK6027	*lst-1(q1008)[lst-1::3xOLLAS] sygl-1(tm5040) I*	*This work*
