# Supplementary material for: SYGL-1 and LST-1 link niche signaling to PUF RNA repression for stem cell maintenance in Caenorhabditis elegans
Source: PLoS Genet. 2017 Dec 12;13(12):e1007121. doi: 10.1371/journal.pgen.1007121 (PMC5741267; doi:10.1371/journal.pgen.1007121)
Supplement: S2 Table — (PDF) [file pgen.1007121.s010.pdf]

S2 Table. MosSCI transgenes generated in this study

Allele	Insert Description	Injected plasmid	Parent strain	Integration locus
qSi22	$P_{lst\text{-}1}$*::lst-1::1xHA::lst-1* 3'end	pJK1631	EG4322	*ttTi5605*
qSi49	$P_{sygl\text{-}1}$*::3xFLAG::sygl-1::sygl-1* 3'end	pJK1658	EG6699	*ttTi5605*
qSi69	$P_{lst\text{-}1}$*::lst-1::3xFLAG::lst-1* 3'end	pJK1692	JK4950	*ttTi5605*
qSi93	$P_{lst\text{-}1}$*::lst-1::1xHA::lst-1 3'end*	pJK1734	EG6703	*cxTi10816*
qSi150	$P_{sygl\text{-}1}$*::3xFLAG::sygl-1::tbb-2* 3'end	pJK1798	JK4966	*ttTi5605*
qSi235	$P_{mex\text{-}5}$*::3xFLAG::sygl-1::tbb-2* 3'end	pJK1873	JK4966	*ttTi5605*
qSi267	$P_{mex\text{-}5}$*::lst-1::3xFLAG::tbb-2* 3'end	pJK1898	JK4950	*ttTi5605*
qSi297	$P_{mex\text{-}5}$*::3xMYC::sygl-1::tbb-2* 3'end	pJK1897	JK4966	*ttTi5605*
