# Supplementary material for: SYGL-1 and LST-1 link niche signaling to PUF RNA repression for stem cell maintenance in Caenorhabditis elegans
Source: PLoS Genet. 2017 Dec 12;13(12):e1007121. doi: 10.1371/journal.pgen.1007121 (PMC5741267; doi:10.1371/journal.pgen.1007121)
Supplement: S3 Table — (PDF) [file pgen.1007121.s011.pdf]

S3 Table. CRISPR alleles generated in this study

Allele	Description	Guide	Repair template	Parent strain	Method
q828	*sygl-1* null mutant	pJK1875 pJK1879 pJK1800	pJK1799	HT1593	Plasmid injection; Dickinson *et al* 2013
q931	*3xV5::fbf-2*	*fbf-2* crRNA N-term	*3xV5 fbf-2* oligo	JK5366	RNP co-CRISPR; Arribere *et al*, 2014, Paix *et al*, 2015
q932	*3xV5::fbf-2*	*fbf-2* crRNA N-term	*3xV5 fbf-2* oligo	JK5574	
q964	*3xMYC::sygl-1*	*sygl-1* crRNA N-term	pJK1926	wild type	
q983	*3xOLLAS::sygl-1*	*sygl-1* crRNA N-term	*3xOLLAS sygl-1* oligo	wild type	
q1004	*lst-1::3xV5*	*lst-1* crRNA C-term	*lst-1 3xV5* oligo	wild type	
q1008	*lst-1::3xOLLAS*	*lst-1* crRNA C-term	*lst-1 3xOLLAS* oligo	wild type	
q1015	*sygl-1::1xV5*	*sygl-1* crRNA C-term	*sygl-1 1xV5* oligo	JK5921[a]	

[a] Aoki ST and Kimble J (in preparation)
