# Supplementary material for: SYGL-1 and LST-1 link niche signaling to PUF RNA repression for stem cell maintenance in Caenorhabditis elegans
Source: PLoS Genet. 2017 Dec 12;13(12):e1007121. doi: 10.1371/journal.pgen.1007121 (PMC5741267; doi:10.1371/journal.pgen.1007121)
Supplement: S4 Table — (PDF) [file pgen.1007121.s012.pdf]

S4 Table. Plasmids used to generate CRISPR and MosSCI transgenes

Plasmid	Insert Description	Cloning sites	Vector Backbone
pJK1631	$P_{lst\text{-}1}$ (~2.6kb)*::lst-1::1xHA::lst-1* 3'end (~0.6kb)	*Bss*H II	pCFJ151
pJK1658	$P_{sygl\text{-}1}$ (~2kb)*::3xFLAG::sygl-1::sygl-1* 3'end (~0.8kb)	*Spe* I	pCFJ151
pJK1692	$P_{lst\text{-}1}$ (~2.6kb)*::lst-1::3xFLAG::lst-1* 3'end (~0.6kb)	*Spe* I	pCFJ151
pJK1734	$P_{lst\text{-}1}$ (~2.6kb)*::lst-1::1xHA::lst-1* 3'end (~0.6kb)	*Xba* I<br>*Bss*H II	pCFJ151
pJK1798	$P_{sygl\text{-}1}$ (~2kb)*::3xFLAG::sygl-1::tbb-2* 3'end (~0.3kb)	*Spe* I	pCFJ151
pJK1799	*sygl-1* upstream homology (~1.6kb)*::loxP::*<br>$P_{unc\text{-}119}$*::Cbr_unc-119::unc-119* 3'UTR*::loxP*::<br>*sygl-1* downstream homology (~1.6 kb)	*Xma* I	pUC19
pJK1873	$P_{mex\text{-}5}$ (~0.5kb)*::3xFLAG::sygl-1::tbb-2* 3'end (~0.3kb)	*Spe* I	pCFJ151
pJK1875	Sequence targeting *sygl-1* loci (5'-agatttcgactaacaactc-3')<br>joined with sgRNA scaffold from pDD162 [a]	*Xma* I	pUC19
pJK1879	Sequence targeting *sygl-1* loci (5'-tttattttcgcaagcacgg-3')<br>joined with sgRNA scaffold from pDD162 [a]	*Xma* I	pUC19
pJK1800	Sequence targeting *sygl-1* loci (5'-gtaactgtggagaccaaat-3')<br>joined with sgRNA scaffold from pDD162 [a]	*Xma* I	pUC19
pJK1898	$P_{mex\text{-}5}$ (~0.5kb)*::lst-1::3xFLAG::tbb-2* 3'end (~0.3kb)	*Spe* I	pCFJ151
pJK1897	$P_{mex\text{-}5}$ (~0.5kb)*::3xMYC::sygl-1::tbb-2* 3'end (~0.3kb)	*Spe* I	pCFJ151
pJK1926	$P_{sygl\text{-}1}$ (~2kb)*::3xMYC::sygl-1::sygl-1* 3'end (~0.8kb)	*Spe* I	pCFJ151

[a] Dickinson *et al* (2013)
