# Supplementary material for: SYGL-1 and LST-1 link niche signaling to PUF RNA repression for stem cell maintenance in Caenorhabditis elegans
Source: PLoS Genet. 2017 Dec 12;13(12):e1007121. doi: 10.1371/journal.pgen.1007121 (PMC5741267; doi:10.1371/journal.pgen.1007121)
Supplement: S5 Table — (PDF) [file pgen.1007121.s013.pdf]

S5 Table. Sequences of crRNA and repair oligos used to generate CRISPR alleles

Name	Sequence[1,2,3]
sygl-1 crRNA N-term	5’-AUGGAAUGGCAUUAUGCACGGUUUUAGAGCUAUGCU-3’
sygl-1 crRNA C-term	5’-CUACUGCAAAUAAUAGCUGUGUUUUAGAGCUAUGC-3’
lst-1 crRNA C-term	5’-UCCAGUCUAAGCAAUAAAAUGUUUUAGAGCUAUGCU-3’
fbf-2 crRNA N-term	5’-UCGUUCUGCGCAUCUUUGAUGUUUUAGAGCUAUGCU-3’
3xOLLAS sygl-1 oligo	5’-gtgatccatgtagagttttggataatggaatggCTTTCCCATAAGGCGTGGTCCGA<br>GCTCGTTGGCGAATCCAGACTGCTTTCCCATGAGGCGTGGTCCGAG<br>CTCGTTGGCGAATCCAGACTGCTTTCCCATAAGGCGTGGTCCAAGC<br>TCGTTAGCGAATCCGGAcattatgcacgtggcgtgatgacaatggttcgatg-3’
sygl-1 1xV5 oligo	5’-gaacaacaacacttcactgatgatgggctc**T**aacagctattatttgcagGGTAAGCCTAT<br>CCCTAACCCTCTCCTCGGTCTAGATAGTACTGGAGGATCCtagagcgta<br>cttgctcttttaaattttctaatcc-3’
lst-1 3xV5 oligo	5’-caaatgggacacgctcgaaatgttccagtcGGTAAGCCTATCCCTAACCCTCTC<br>CTCGGTCTAGATAGTACTGGAAAGCCAATCCCAAACCCACTCCTCG<br>GACTTGATAGCACCGGTAAGCCTATCCCTAACCCACTCCTCGGACTT<br>GATAGCACCtaagcaataaaattggtttaaatatcaattaatttatattttac-3’
lst-1 3xOLLAS oligo	5’-tataaattaattgatatttaaaccaattttattgcttaCTTTCCCATAAGGCGTGGTCCG<br>AGCTCGTTGGCGAATCCAGACTGCTTTCCCATGAGGCGTGGTCCGA<br>GCTCGTTGGCGAATCCAGACTGCTTTCCCATAAGGCGTGGTCCAAG<br>CTCGTTAGCGAATCCGGAgactggaacatttcgagcgtgtcccatttg-3’
3xV5 fbf-2 oligo	5’-tcattctaataaaattatcaactaatcgacatgGGTAAGCCTATCCCTAACCCTCT<br>CCTCGGTCTAGATAGTACTGGAAAGCCAATCCCAAACCCACTCCTC<br>GGACTTGATAGCACCGGTAAGCCTATCCCTAACCCACTCCTCGGAC<br>TTGATAGCACCga**T**caatcaaagatgcgcagaacgaatcagttcagaaaagt-3’

1. Upper case letters indicate inserted sequences
2. Lower case letters indicate homology arms
3. **Bold** letters indicate mutations incorporated
